# Reproductive fluids, commercial media, and organoids: bridging the gap in IVF culture systems

**DOI:** 10.1093/hropen/hoag028

**Published:** 2026-04-02

**Authors:** Nathaly Hernández-Díaz, Sergio Navarro-Serna, Pilar Coy, Vicente Pérez-García

**Affiliations:** Centro de Biología Molecular Severo Ochoa, CSIC-UAM, Madrid, Spain; Department of Physiology, University of Murcia, Campus of Excellence Mare Nostrum, IMIB-Arrixaca Pascual Parrilla, Murcia, Spain; Centro de Biología Molecular Severo Ochoa, CSIC-UAM, Madrid, Spain; Centro de Investigación Príncipe Felipe, Valencia, Spain; Department of Physiology, University of Murcia, Campus of Excellence Mare Nostrum, IMIB-Arrixaca Pascual Parrilla, Murcia, Spain; Centro de Biología Molecular Severo Ochoa, CSIC-UAM, Madrid, Spain

**Keywords:** ART, embryo culture media, human IVF, reproductive fluids, endometrial organoids, oviductal organoids, extracellular vesicles, endometrial receptivity, implantation failure, biomimetic systems

## Abstract

**BACKGROUND:**

Although assisted reproductive technologies have enabled millions of births worldwide, *in vitro* embryo culture systems remain a simplified and static approximation of the highly dynamic environment of the female reproductive tract. Commercial culture media lack many of the biochemical, biophysical, and temporal features of oviductal and uterine fluids, including hormonally regulated secretions, extracellular vesicles, and epithelial-derived signaling cues. These limitations are increasingly linked to altered embryonic programming, suboptimal implantation, and subtle but persistent effects on perinatal and long-term health outcomes.

**OBJECTIVE AND RATIONALE:**

This review critically examines how reproductive tract-derived factors and advanced three-dimensional (3D) *in vitro* models can improve the physiological relevance of embryo culture systems in human ART. We focus on the biological roles of native reproductive fluids and extracellular vesicles and the emerging contribution of reproductive tract organoids and assembloids as sources of defined, stage-specific secretomes capable of bridging the gap between artificial and *in vivo*-like conditions.

**SEARCH METHODS:**

A comprehensive literature search was conducted in PubMed, Scopus, and Web of Science for studies published up to March 2026 using terms related to embryo culture media, reproductive fluids, extracellular vesicles, organoids, implantation, and human IVF. Evidence from human studies and relevant animal models was included to provide mechanistic insight and translational context, with emphasis on experimental approaches directly informing ART practice.

**OUTCOMES:**

Reproductive tract fluids contain complex mixtures of proteins, metabolites, lipids, and extracellular vesicles that regulate fertilization, early embryonic development, epigenetic programming, and maternal–embryo communication. While supplementation of embryo culture media with native fluids improves embryo quality and developmental competence in multiple species, clinical translation is constrained by donor variability, biosafety concerns, and limited standardization. Reproductive tract 3D cell cultures represent a promising complementary approach, as they can recapitulate key aspects of epithelial architecture, hormonal responsiveness, and secretory activity under controlled conditions. Organoid-derived secretomes, including extracellular vesicle cargo, have been shown to support reproductive processes such as sperm viability, trophoblast function, immune modulation, and endometrial receptivity. Moreover, advances in epithelial-stromal assembloids and microengineered platforms further enhance physiological fidelity by partially restoring multicellular interactions relevant to implantation-related signaling. However, these systems also present important limitations, including variability between lines, incomplete cellular complexity, scalability challenges, and unresolved regulatory considerations for clinical translation.

**LIMITATIONS, REASONS FOR CAUTION:**

This review is based on heterogeneous evidence derived from both human and animal studies, which may limit direct clinical translation due to species-specific differences in reproductive physiology and embryo development. Variability in experimental design, culture conditions, and reporting standards across studies introduces potential bias and complicates comparative interpretation. In particular, the use of reproductive fluids is subject to significant inter- and intra-donor variability, differences in collection and processing methods, and incomplete biochemical characterization, all of which represent important confounding factors. Similarly, organoid and assembloid models exhibit variability between lines, incomplete cellular complexity, and differences in differentiation state, which may influence secretome composition and functional outcomes. Moreover, many studies have relied on surrogate endpoints, such as embryo morphology or blastocyst formation, rather than long-term clinical outcomes, limiting conclusions regarding safety and efficacy in human ART.

**WIDER IMPLICATIONS:**

Organoid- and assembloid-derived secretomes represent a scalable, ethically sustainable, and mechanistically tractable strategy to advance biomimetic embryo culture in human ART. These systems provide a framework for defining biologically relevant secretory profiles, enabling stage-specific and, potentially, patient-informed supplementation strategies. Integrating reproductive tract organoid technologies with extracellular vesicle biology and dynamic culture platforms may ultimately improve embryo competence, implantation success, and long-term offspring health, while supporting safer and more physiologically informed ART practices.

**STUDY FUNDING/COMPETING INTEREST(S):**

This work was funded by the Ministerio de Ciencia e Innovación and the Agencia Estatal de Investigación (MICIU/AEI/10.13039/501100011033) under grant numbers PLEC2022-009246, PID2023-148535OB-I00, and CNS2022-135933; the European Social Fund (ESF), Investing in Your Future; and the Fundación Ramón Areces. N.H.-D. is supported by a Marie Curie PhD fellowship from the AFRODITA Doctorate Network, funded by the HE programme under the MSCA-DN grant agreement No. 101120126. None of the authors have a conflict of interest to disclose.

WHAT DOES THIS MEAN FOR PATIENTS? IVF has helped millions of people have children, but embryos are grown in laboratory conditions that do not fully match the natural environment inside the body. In the female reproductive tract, early embryos are exposed to a complex and changing mix of fluids and signals that support their development. Current IVF culture systems cannot fully reproduce these conditions. This review describes new approaches to make embryo culture more similar to natural conditions. One strategy is to use components from reproductive fluids, which contain important molecules that guide early development. Another approach uses advanced laboratory models, called organoids, which are small three-dimensional tissue structures that can produce these supportive factors in a controlled way. These innovations may improve embryo development, implantation, and pregnancy outcomes and could support healthier babies. However, further research is needed to confirm their safety and effectiveness before they can be widely used in clinical practice.

## Introduction

Current IVF culture systems support acceptable early embryo development but fail to reproduce the dynamic biochemical, mechanical, and temporal cues of the reproductive tract (Sciorio and Rinaudo, 2023). *In vivo*, preimplantation embryos are exposed to a continuously changing environment shaped by epithelial secretions, mucins, glycoproteins, extracellular vesicles (EVs), fluid flow, and hormonal oscillations (Kölle *et al.*, 2009; Godakumara *et al.*, 2023). These factors regulate metabolic activity, epigenetic remodeling, developmental timing, and maternal–embryo communication (Pavličev *et al.*, 2017). In contrast, *in vitro* culture (IVC) media still rely on static media that lack many of these features, offering only a partial approximation of physiological conditions (Cairo Consensus Group, 2020).

These discrepancies between *in vivo* and *in vitro* conditions contribute to measurable differences in metabolic programming, transcriptional profiles, and fetal growth trajectories between *in vivo*- and *in vitro*-derived conceptuses (Aksu *et al.*, 2012; Driver *et al.*, 2012). Such limitations underscore the need for more biomimetic culture systems capable of integrating essential biochemical and biophysical properties of reproductive tract fluids. Advances in reproductive tract organoids and assembloids, as well as their secretome, now provide new opportunities to generate defined, reproducible, and physiologically relevant supplements that may overcome the constraints associated with native reproductive fluids.

This review synthesizes the current evidence on reproductive fluids, commercial media, and three-dimensional (3D) cell culture-derived factors, highlighting conceptual advances, methodological limitations, and the translational challenges of implementing physiologically inspired culture systems.

## Culture media: current performance and intrinsic limitations

Assisted reproductive technologies have resulted in more than 10 million births since 1978 (Edwards *et al.*, 1969, 1970; Adamson *et al.*, 2025; Baker *et al.*, 2025). Contemporary culture media incorporate salts, carbohydrates, amino acids, buffers, and protein supplements but lack many critical components and physical properties characteristic of reproductive tract fluids (Sunde *et al.*, 2016; Zagers *et al.*, 2025).

Since the earliest mammalian media (e.g. Earle’s, Bavister’s, Waymouth’s, Whittingham’s), human embryo culture formulations have been progressively refined into modern sequential and single-step systems (Edwards *et al.*, 1970; Morbeck *et al.*, 2017; Zagers *et al.*, 2025). Yet, no consensus exists on a superior medium, and reported differences in clinical or laboratory outcomes are often modest and inconsistent. Meaningful comparison is further hampered by proprietary compositions, divergent protein supplements, variable oxygen tension, and non-standardized laboratory practices, which together limit mechanistic interpretation (Sciorio and Rinaudo, 2023).

Contemporary media are broadly categorized as chemically defined or undefined, depending on their protein source. Defined formulations offer greater reproducibility and quality control, whereas undefined supplements, such as serum or reproductive fluids, introduce inter- and intra-donor variability that complicates standardization. In general, commercial media combine inorganic salts, carbohydrates, amino acids, buffers, and protein supplements, with optional additions such as antibiotics, vitamins, and chelators (Table 1). However, the precise contribution of individual components to embryo competence remains difficult to disentangle, and authors have therefore called for full transparency in media composition and patient cohorts (Sunde *et al.*, 2016; Tarahomi *et al.*, 2019; Zagers *et al.*, 2025). Even under optimized conditions, these formulations force embryos to adapt to an artificial environment and to activate metabolic and molecular stress responses to maintain development *in vitro* (Lee *et al.*, 2022; Zhang *et al.*, 2025).

**Table 1.** Composition of selected commercial *in vitro* embryo culture media compared with reference basal media.

Brand				Fujifilm Irvine Sci.	Vitrolife	Vitrolife	Vitrolife	Cooper Surgical	Cooper Surgical	Cooper Surgical	Gynemed	FertiPro
**Medium**	Modified Earle’s	Mouse Egg Medium	Ham’s F10	CSCM-NXC	G-TL™	G-1™ PLUS	G-2™ PLUS	SAGE 1-Step™ GM-CSF	EmbryoGen^®^	BlastGen™	GM501 Cult with Gentamicin	GAIN™ Medium
**Culture protocol**				One step	One step	Sequential	Sequential	One step	Sequential	Sequential	One step	One step
**Salts**												
CaCl_2_	×		×	×	×	×	×	×	×	×	×	×
KCl	×	×	×	×	×	×	×	×	×		×	×
KH_2_PO_4_		×									×	
MgSO_4_	×	×	×	×	×	×	×	×	×	×	×	×
Na_2_HPO_4_			×									×
NaCl	×	×	×	×	×	×	×	×	×	×	×	×
NaH_2_PO_4_	×			×	×	×	×	×	×	×	×	
NaHCO_3_	×	×	×	×	×	×	×	×	×	×	×	×
**Carbohydrates**												
Calcium lactate		×										×
Glucose	×	×	×	×				×	×	×	×	×
Sodium citrate	×			×	×	×	×	×	×	×		
Sodium lactate				×	×	×	×	×	×	×	×	
Sodium pyruvate	×	×	×	×	×	×	×	×	×	×	×	×
**Amino acids and protein source**												
Essential amino acids				×	×	×	×	×	×	×	×	
Human serum albumin				×	×	×	×	×	×	×	×	×
Hyaluronic acid				×	×	×	×	×	×	×		
Non-essential amino acids				×	×	×	×	×	×	×	×	
Taurine									×			
**Others**												
EDTA				×	×	×	×	×	×	×	×	×
Vitamins				×	×		×	×	×	×		
Lipoic acid (antioxidant)				×	×	×						
Additional antioxidants				×	×		×	×	×	×		
Gentamicin (antibiotic)					×		×	×	×	×	×	×
GM-CSF (cytokine)								×	×	×		

× indicates the presence of the component in the formulation according to manufacturer descriptions. Commercial media compositions are based on publicly available information and may not reflect proprietary or undisclosed components. Culture protocols are classified as one-step (single medium throughout development) or sequential (stage-specific media). CaCl_2_, calcium chloride; KCl, potassium chloride; KH_2_PO_4_, potassium dihydrogen phosphate; MgSO_4_, magnesium sulfate; Na_2_HPO_4_, disodium hydrogen phosphate; NaCl, sodium chloride; NaH_2_PO_4_, sodium dihydrogen phosphate; NaHCO_3_, sodium bicarbonate; EDTA, ethylenediaminetetraacetic acid; GM-CSF, granulocyte–macrophage colony-stimulating factor.

## The influence of culture media on embryonic programming

Embryo culture medium performance is commonly evaluated based on early developmental parameters such as cleavage rate, cell division kinetics, and blastocyst formation. However, implantation and gestational success, typically measured by live birth rate, remain the most meaningful clinical endpoint (Gnoth *et al.*, 2011). The period of *in vitro* embryo culture coincides with the global epigenetic reprogramming of the preimplantation embryo (Zhu *et al.*, 2018). This developmental window is therefore considered particularly sensitive to environmental influences. Despite this biological susceptibility, relatively few studies have directly assessed the specific impact of culture media on the epigenome of embryos and extraembryonic tissues.

So far, the direct effect of IVC conditions on placental DNA methylation (DNAm) is modest and inconsistent across cohorts, with only a limited number of recurrent candidate loci. Analysis of umbilical cord blood (UCB) from neonates conceived under different culture media (G5, Vitrolife; human tubal fluid (HTF) medium, HTF, Lonza) shows no differences in DNAm between media groups (Koeck *et al.*, 2022). However, when UCB from ART-conceived newborns is compared with that of naturally conceived (NC) newborns, global DNA hypomethylation is observed (Håberg *et al.*, 2022). Similarly, ART-derived term placentas exhibit less DNAm at imprinted loci such as the *PEG1/MEST* region (Nelissen *et al.*, 2013; Barberet *et al.*, 2022), alongside downregulation of *TRIM28*, a key stabilizer of genomic imprinting (Auvinen *et al.*, 2024).

Beyond methylation patterns, transcriptomic analyses indicate that ART-associated placental changes are enriched in pathways related to hormonal regulation, insulin secretion, and vascular development, particularly through downregulation of *NOTCH3* and *DLK1* genes (Auvinen *et al.*, 2024). Whether these molecular signatures directly translate into apparent structural placental abnormalities, such as accelerated villous maturation, placenta previa, placental abruption, or placenta accreta spectrum, remains ambiguous (Vermey *et al.*, 2019; Londero *et al.*, 2021; Matsuzaki *et al.*, 2021).

Clinically, although children conceived through ART are generally healthy, the aforementioned molecular findings, together with epidemiological evidence, offer a plausible mechanistic link of ART with an increased risk of cardiometabolic and vascular disorders, imprinting disorders, preeclampsia, hypertensive complications of pregnancy, and low birth weight (Qin *et al.*, 2017; Cui *et al.*, 2020; Pinborg *et al.*, 2023; Auvinen *et al.*, 2024). Birth weight, as a representative outcome, reflects a complex interplay of parental, embryological, and genetic determinants. Within this multifactorial framework, growing evidence suggests that embryo culture media formulation and culture strategy may contribute measurably to perinatal growth patterns, even if their molecular effects are subtle and context-dependent (Ahlström *et al.*, 2023; Sonigo *et al.*, 2024; Li *et al.*, 2025).

For instance, independent and randomized studies comparing human embryo culture mediums, including GI.3, G5, G5-plus (Vitrolife), and HTF versus K-SICM (Cook) medium, showed that singletons cultured using Vitrolife and HTF mediums had higher birth weights (Dumoulin *et al.*, 2010; Li *et al.*, 2025). In fact, Vitrolife versus Cook culture medium growth differences persisted through the first 2 years of life, highlighting how subtle differences in media composition can have lasting postnatal effects (Kleijkers *et al.*, 2014). Further examples include mitochondrial DNA (mtDNA) variants, which in NC individuals are associated with body weight (Flaquer *et al.*, 2014). This relationship appears only partially preserved in ART cohorts, as an enrichment of *de novo* mtDNA mutations (particularly non-synonymous and rRNA variants, also present in NC groups) seems to be magnified in ART populations, with additional influence from maternal age, ovarian stimulation, and IVC-related factors (Mertens *et al.*, 2024). In this case, exposure to Cook or UZB (an in-house medium made in the UZ Brussel) embryo culture medium appears to override the effect of mtDNA variation on fetal growth, whereas embryos cultured in Vitrolife medium show similar patterns to those of the NC groups (Mertens *et al.*, 2024).

Importantly, current findings underscore that ART-associated molecular signatures are not yet universal but vary across the populations, study designs, and developmental stages assessed. The lack of standardization in procedural variables (e.g. media type, oxygen conditions, IVF/ICSI embryo transfer protocols) across clinics and even within the same institution creates inconsistencies that can mask subtle biological effects and challenge the interpretation of comparative analyses (Racowsky *et al.*, 2026). These limitations also reinforce the broader conceptual shift required in the field: moving beyond merely “supportive” culture environments toward culture systems that are actively biomimetic, capable of recapitulating the biochemical, biophysical, and temporal complexity of the reproductive tract.

This focus is increasingly driving interest in the use of reproductive-fluid supplementation, dynamic co-culture platforms, and organoid or assembloids-derived secretomes, all of which aim to reintroduce aspects of the physiological niche into embryo culture. Their integration with 3D-cell culture models and embryo co-culture models represents a key frontier in constructing more faithful reproductive microenvironments, and insights from these platforms will likely inform the next generation of biomimetic media.

## Reproductive fluids as biomimetic additives: a model for optimized media?

**Figure 1.**
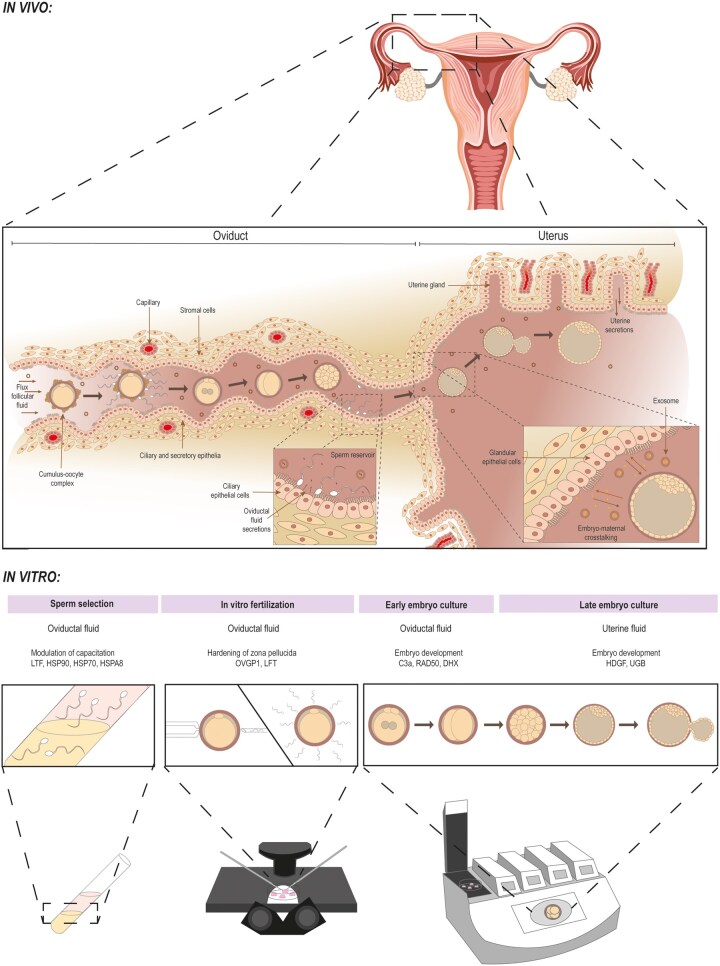
**Comparison of fertilization and early embryo development in the female reproductive tract and in laboratory culture conditions.** The upper panel illustrates fertilization and preimplantation embryo development *in vivo* within the female reproductive tract. In this physiological context, gametes and embryos are exposed to a dynamic and complex microenvironment composed of follicular, oviductal, and uterine fluids. These fluids contain a wide range of bioactive components, including proteins, metabolites, lipids, hormones, and extracellular vesicles, and are influenced by epithelial secretions, fluid flow, and hormonal changes. Together, these factors regulate fertilization, early embryonic development, and maternal–embryo communication. The lower panel depicts the conventional IVF workflow, in which sperm preparation, fertilization, and embryo culture are performed using static, chemically defined culture media. These systems lack many of the biochemical and physical properties of the *in vivo* environment. The figure highlights how supplementation with reproductive fluids, or their bioactive components, at specific stages of *in vitro* culture may improve fertilization outcomes and embryo quality by more closely mimicking physiological conditions. IVP, *in vitro* embryo production.

Replicating the *in vivo* environment in *in vitro* embryo culture is by far more than a simple combination of salts, energy substrates, and amino acids (Fig. 1). Modern approaches emphasize the need to adapt the composition of the culture media to the specific metabolic and developmental needs of the embryo at each stage. This includes not only adjusting inorganic elements but also incorporating a wide range of organic compounds (vitamins, lipids, growth factors, and antioxidants), many of which are naturally present in oviductal and uterine fluids (UFs) (Table 2). Analyses of human oviductal fluid (OF) revealed substantial discrepancies in amino acids, energy substrates, and ionic composition when compared with conventional media, supporting the development of oviduct-inspired formulations such as OVIT (Utsunomiya *et al.*, 2022).

**Table 2.** Concentrations of major osmolytes in human uterine fluid, serum, and oviductal fluid across reproductive phases.

	Uterine fluid			Serum	Oviductal fluid	Oviductal fluid	
	Proliferative phase	Mid-cycle phase	Luteal phase			Preovulatory phase	Postovulatory phase
Osmolarity (mOsm/kg)	276–307	276–298	282–302	278–302	–	–	–
K (mmol/l)	24.5–27.5	12.6–25.7	18.8–28.0	3.7–4.8	3.6–5.0	9.9 ± 1.8	7.7 ± 0.9
Na (mmol/l)	98–118	96–130	99–119	139–145	139–147	140 ± 3	139 ± 2
Ca (mmol/l)	0.41–1.98	0.21–1.69	1.01–2.49	2.29–2.85	2.27–2.72	1.89 ± 0.52	2.37 ± 0.27
Cl (mmol/l)	108–111	105–117	111–117	105‒114	102–113	119 ± 4	117 ± 3

Values are reported as ranges or mean ± standard deviation, depending on the original study. Uterine fluid values correspond to different phases of the menstrual cycle (proliferative, mid-cycle, and luteal). Oviductal fluid values correspond to preovulatory and postovulatory stages. Ca, calcium; Cl, chloride; K, potassium; Na, sodium; mOsm/kg, milliosmoles per kilogram. Data sources: Casslén and Nilsson, (1984); Borland *et al*. (1980); Lippes *et al*. (1972).

Supplementation of media for *in vitro* oocyte maturation, fertilization, and embryo culture may serve as a foundation for next-generation approaches aimed at biomimicking natural reproductive fluids (Canovas *et al.*, 2017; París-Oller *et al.*, 2021), although their ability to emulate the biochemical and physical conditions of the oviduct and uterus remains limited. *In vivo*, embryos experience a dynamic microenvironment characterized not only by temporally regulated nutrient concentrations but also by fluid flow, ciliary movement, mucus-rich secretions, and complex interactions with epithelial surfaces (Coy *et al.*, 2012). Current culture media compensate only partially for these attributes, often lacking immunoregulatory glycoproteins, cytokines, EVs, and the dynamic fluctuations of ions and amino acid profiles that characterize the *in vivo* uterine environment (Zagers *et al.*, 2025).

Reproductive tract fluids contain thousands of components, including proteins, glycoproteins, lipids, metabolites, EVs, cytokines, miRNAs, and hormones, which contribute to the embryo’s natural microenvironment (Li *et al.*, 2023; Piibor *et al.*, 2023; Gonella-Diaza *et al.*, 2024; Apostolov *et al.*, 2025). Unsurprisingly, numerous studies have explored the use of reproductive fluids or their derivatives as supplements to the medium as a strategy to improve biomimicry *in vitro* (Canovas *et al.*, 2017; París-Oller *et al.*, 2021). Studies in livestock demonstrate that reproductive fluids can influence fertilization, cleavage, gene expression, metabolic activity, placental vascularization, and pregnancy rates, although outcomes vary markedly across species, experimental conditions, and fluid origin (París-Oller *et al.*, 2022; Párraga-Ros *et al.*, 2023; Heras *et al.*, 2025b). These inconsistencies highlight both the promise and the complexity of using native physiological secretions to support embryo development.

## Oviductal and uterine reproductive fluids in embryo culture

OF provides a dynamic milieu shaped by ciliary activity, epithelial secretion, and endocrine fluctuations. Before fertilization, OF undergoes key modifications that facilitate gamete interaction and fertilization, e.g. oviduct-specific glycoprotein 1 (OVGP1) promotes zona pellucida hardening in polyspermy-prone species, such as pigs and goats, thereby reducing fertilization anomalies (Coy *et al.*, 2008; Bragança *et al.*, 2021). Because preimplantation development naturally spans two distinct environments (the oviduct during cleavage and the uterus during compaction and blastocyst formation), several studies have explored sequential supplementation strategies (Hamdi *et al.*, 2018; Leal *et al.*, 2022; Pakniyat *et al.*, 2025). IVC of porcine embryos, first in OF (1% v/v) and then in UF (1% v/v), produced blastocysts that more closely resembled their *in vivo* counterparts in cell number and epigenetic signatures (Canovas *et al.*, 2017; París-Oller *et al.*, 2021). Follow-up analyses in this porcine model revealed that supplementation with reproductive fluids also mitigated aberrant placental gene expression typically associated with *in vitro* embryo production. Placentas derived from embryos cultured with reproductive fluids did not show the increased expression of developmentally relevant genes such as *PEG3* and *LUM*, which were otherwise dysregulated in conventional (IVC) conditions, suggesting that fluid-derived factors contribute to early placental programming with downstream consequences for offspring development (París-Oller *et al.*, 2021).

Human reproductive fluids collected under well-established quality control protocols demonstrated bioactivity in a bovine embryo assay, with 1% (v/v) supplementation of culture medium supporting high cleavage rates and blastocyst formation. As a subsequent proof-of-concept in this study, embryos cultured with autologous UF could develop into successful pregnancies in three women, with two of them resulting in healthy live births (Canha-Gouveia *et al.*, 2021).

Despite this evidence, responses to reproductive fluids are species-specific and dose-dependent, as excessive concentrations of reproductive fluids can impair development. This is relevant in cattle embryos, where the use of oviductal and UFs during IVC in more than 5% of cases was negative (Hamdi *et al.*, 2018). Similarly, exposure of mouse oocytes/cumulus complexes to ovarian endometriotic fluid led to a significant reduction in the proportion of hatching/hatched blastocysts, even when initial fertilization and cleavage rates were not substantially altered (Piromlertamorn *et al.*, 2013). These findings highlight that defining supplementation merely as a percentage (v/v) may be insufficient, as protein composition and concentration can vary substantially between fluid batches. Future strategies should therefore aim to standardize supplementation based on quantitative characterization of key protein and bioactive component concentrations, enabling tighter control of exposure levels and minimizing the risk of toxicity or developmental perturbations.

Overall, reproductive fluids and their bioactive components provide a valuable baseline for designing physiologically relevant culture systems. When incorporated in a controlled and standardized way, these components have the potential to enhance embryo quality and developmental progression, supporting improved outcomes in ART (Lopes *et al.*, 2020; Heras *et al.*, 2025a,b; Serrano-Albal *et al.*, 2025). However, the direct use of biological fluids poses potential biosafety risks, including the transmission of infectious agents, inflammatory mediators, and immunogenic components. Translating approaches that incorporate reproductive fluids as standard supplements for IVC systems would require addressing stringent safety regulations and donor-screening requirements. Moreover, only a limited number of studies have demonstrated the feasibility of implementing such strategies in a controlled and reproducible manner.

## EVs: emerging mediators of reproductive communication

EVs have been isolated from a wide range of reproductive sources (e.g. human, murine, livestock), including follicular fluid, OF, UF, embryo-conditioned media, and oviduct epithelial cell cultures, underscoring their central role in maternal–embryo communication (Ma *et al.*, 2022; Li *et al.*, 2023; Hamdi *et al.*, 2024; Muraoka *et al.*, 2024; Pakniyat *et al.*, 2025). These vesicles transport bioactive cargo, such as miRNAs, proteins, and membrane receptors, that can be internalized by gametes and embryos, influencing preimplantation development, developmental competence, cell allocation, oxidative balance, and implantation-related pathways (Ma *et al.*, 2022; Li *et al.*, 2023).

Evidence from animal models further supports their function as dynamic mediators of maternal signaling, modulating embryonic development in a stage, dose, and tissue-specific manner. For instance, murine embryos co-cultured with varying doses of human fallopian tube (FT)-derived EVs (from patients with uterine fibroids) showed improved developmental outcomes, although the effects were not strictly dose-dependent nor consistently linked to a particular donor (Li *et al.*, 2023).

Consistent with the concept of temporally regulated EV signaling, murine oviduct-derived EVs isolated across estrous stages enhance blastocyst yield, cell number, and embryo quality, with the strongest effects in EVs isolated from the diestrus group (representing the highest levels of estrogen receptor, a factor known to support early embryogenesis (Xue *et al.*, 2025). Importantly, when sequential and EV replenishment supplementation was done (particularly on days 3 and 4 post-fertilization), an increase in hatching rates was observed. This suggests that EV bioactivity declines in static culture, and temporal renewal more closely mimics the continuous *in vivo* exposure within the reproductive tract (Xue *et al.*, 2025).

While isolated studies in bovines have reported that specific EV-associated components, such as miR-146b, present in conditioned culture medium, may exert detrimental effects on embryos (Pavani *et al.*, 2024), the broader body of evidence supports a predominantly beneficial role of reproductive EVs in early development. Studies using medium supplemented with microRNA (miR-378a-3p from embryonic sources) and follicular oviductal- and UF-derived EVs support an overall increase in blastocyst yield and hatching rates (Hamdi *et al.*, 2018, 2024; Pavani *et al.*, 2022; Benedetti *et al.*, 2024) and, furthermore, enhanced the expression of embryo development and implantation-relevant genes such as *IFNT*, the key cytokine required for maternal recognition of pregnancy (Leal *et al.*, 2022; Benedetti *et al.*, 2024).

Bovine and murine findings provide a compelling proof of concept, which is further supported by human data in which microRNAs were dysregulated in women with recurrent implantation failure, and were associated with pathways governing endometrial receptivity and implantation competence (Liu *et al.*, 2021a). In this case, endometrial EVs from fertile women improved implantation competence compared with EVs from women experiencing recurrent implantation failure (Liu *et al.*, 2020). These findings support the concept that fluid-derived microRNAs function not only as biomarkers of reproductive outcome but also as integral regulatory signals within the implantation microenvironment.

## Beyond supplementation: toward automated, dynamic, and biomimetic platforms

Early studies showed that the co-culture of human gametes or embryos with reproductive tract epithelial cells enhanced developmental outcomes (Bongso *et al.*, 1989; Yeung *et al.*, 1992). These systems aimed to restore the dynamic, bidirectional communication between the embryo and its maternal environment: interactions that are otherwise absent in conventional static culture. Although traditional co-culture systems are no longer used in routine ART due to advances in next-generation media, they have re-emerged as physiological bioreactors to investigate the maternal–embryo crosstalk and uncover molecular signals lost in simplified media.

This renewed appreciation for the importance of dynamic maternal signaling prompted the development of advanced microfluidic reproductive platforms. Oviduct or uterus-on-a-chip models represent the next conceptual step, offering dynamic control over fluid flow, spatiotemporal gradients, compartmentalization, and luminal shear forces (Ferraz *et al.*, 2018; Ahn *et al.*, 2021; Wang *et al.*, 2022), all of which are features impossible to recreate in conventional IVC drops. Dual chamber “endometrium-on-a-chip” devices, for instance, maintain long-term co-culture of stromal and endothelial cells, enabling decidualization patterns while simulating a full 28-day hormonal cycle (Gnecco *et al.*, 2017). More recent iterations incorporate epithelial, stromal, endothelial, or myometrial compartments in configurations that reproduce the receptivity marker expression and transcriptomic fidelity of native tissue (Ahn *et al.*, 2021; Busch *et al.*, 2024). Collectively, these platforms begin to approximate patient-specific, multi-cellular uterine microenvironments for functional interrogation of embryo–maternal crosstalk (Lee *et al.*, 2025).

Although the application of microfluidic systems to clinical ART remains largely exploratory, these technologies outline what the next generation of IVF platforms could become: dynamic, automated, and biomimetic. The parallel outset of robotic micromanipulation further illustrates this trajectory. The recent successful births following automated ICSI (ICSIA) demonstrate that automated systems can achieve clinical-grade precision, even when operated by individuals without extensive embryology training (Costa-Borges *et al.*, 2023; Mendizabal-Ruiz *et al.*, 2025). The convergence of such robotic technologies and microfluidic culture devices suggests a future in which embryo handling, gamete injection, and early development occur within tightly controlled integrated platforms that function simultaneously as culture systems, physiological bioreactors, and precision instrumentation. Together, they offer a path toward standardizing embryo culture conditions, mitigating procedural heterogeneity (e.g. human-handling, lab-specific practices), one of the most significant sources of inconsistency in ART, and enabling the development of universally applicable protocols across laboratories and clinics (Racowsky *et al.*, 2026).

## Challenges associated with the use of reproductive fluids in ART

Technical limitations, particularly the difficulty of collecting sufficient volumes of oviductal and UF from individual donors, often necessitate pooling samples, thereby introducing inter-individual variability that may mask specific biological effects. Even in controlled animal models, differences in donor physiology, collection methods, storage conditions, and processing pipelines yield fluids with markedly different biochemical profiles and biological effects. Such variability is incompatible with the strict standardization required for human ART. From a regulatory standpoint, reproductive fluids would likely be classified as complex human-derived biological products, necessitating good manufacturing practice (GMP)-compliant procurement, pathogen screening, sterility testing, endotoxin validation, and full traceability: requirements that currently lack standardized implementation pathways in ART laboratories.

Another unresolved key issue is what the authors mean when they state that a certain percentage of “native” or “physiological” reproductive fluid is added as a supplement to the culture media. These additions are often described only in volumetric terms (e.g. 1–10% fluid supplementation), and the biochemical concentration and composition of the supplemented solution remain unknown. Without defining which molecular components are biologically active, at what effective concentrations, and their functional integrity (e.g. mRNA may degrade rapidly under the culture conditions), researchers cannot determine which molecules mediate the observed effect (Chronopoulou and Harper, 2015). Hence, the field would benefit from the establishment of standardized reporting frameworks requiring biochemical characterization, batch qualification criteria, and absolute molecular quantification before clinical translation.

A rational translational pathway may involve deconstructing reproductive fluids into defined molecular components and reconstructing synthetic, quality-controlled mimetics that retain biological functionality while meeting regulatory and safety standards. In this sense, the emergence of 3D tissue cultures and systems offers a captivating alternative. As 3D technology continues to evolve, it holds the potential to redefine IVF and IVC systems by providing this biomimetic microenvironment that closely mirrors the *in vivo* reproductive tract, ultimately improving embryo quality and implantation outcomes in a clinically applicable and ethically sustainable manner.

## Organoids in reproductive research: current advances in 3D *in vitro* models

Organoid technology has revolutionized the study of tissue-specific biology, offering unprecedented opportunities for modeling reproductive tract function *in vitro*. The field was established by seminal work from Hans Clevers’s laboratory, which demonstrated that adult stem cells can generate self-organizing 3D intestinal structures *in vitro* that recapitulate key features of the native tissue, (Sato *et al*., 2009). This foundational breakthrough paved the way for organoid generation from almost all tissues, from a variety of sources of cell types (embryonic, adult, or differentiated cells).

**Figure 2.**
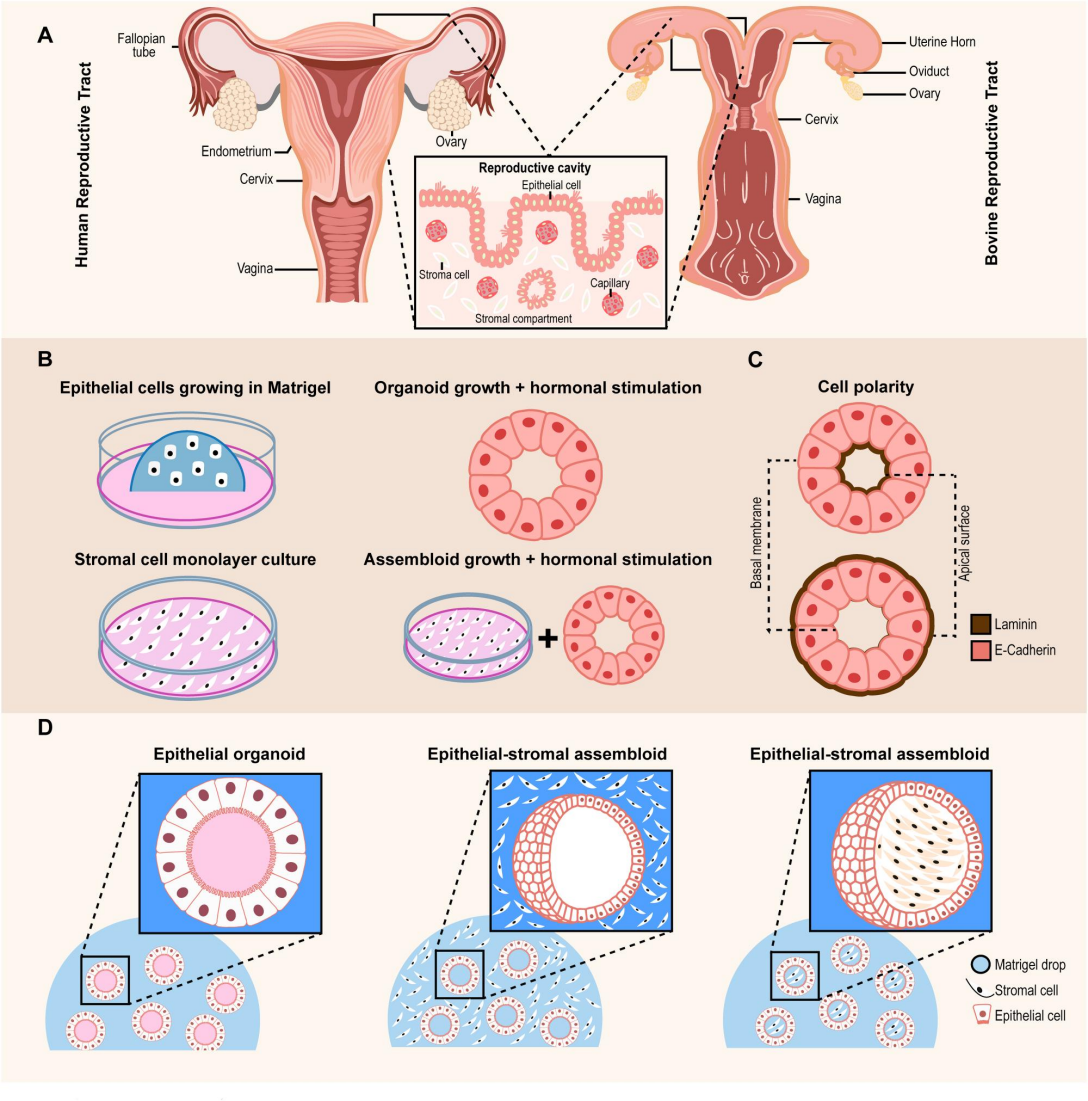
**Generation and structural features of endometrial organoids and assembloids derived from reproductive tissues.** (**A**) Schematic representation of the female reproductive tract in human and bovine species, including the ovaries, fallopian tubes (oviducts), uterus, and cervix. The inset highlights the endometrium, showing the luminal epithelial layer and the underlying stromal compartment. (**B**) Overview of the generation of *in vitro* three-dimensional models. Epithelial cells are isolated and embedded within an extracellular matrix scaffold to form epithelial organoids. Stromal cells can be cultured separately as a monolayer or combined with epithelial cells to generate multicellular epithelial-stromal assembloids that partially recapitulate tissue organization. (**C**) Diagram of epithelial cell polarity within organoids, illustrating apical–basal organization. The apical (luminal-facing) surface is oriented toward the organoid lumen, while the basolateral surface interacts with the surrounding extracellular matrix. (**D**) Representative morphology of epithelial organoids and epithelial-stromal assembloid-like structures grown in extracellular matrix, showing three-dimensional architecture and cellular organization.

Current advances enabled the derivation of oviductal and uterine organoids from a wide range of species, including humans, mice, cattle, pigs, and other domestic animals (Bourdon *et al.*, 2021; Thompson *et al.*, 2023). Resembling native epithelia (Fig. 2A), these organoids comprise at least two cell types: ciliated and non-ciliated or secretory cells (Boretto *et al.*, 2017; Turco *et al.*, 2017). In general, these fundamental traits are critically dependent on active Wnt and Notch signaling pathways (Sato *et al.*, 2011). Accordingly, culture conditions are typically supplemented with growth factors such as epidermal growth factor (EGF), Noggin, and the Wnt agonist R-spondin 1 (RSPO1). Where Wnt signaling (through LGR4/5/6 receptors) maintains epithelial stemness, Notch signaling modulates cell fate, and Noggin prevents premature differentiation (Sato *et al.*, 2011; Mahe *et al.*, 2013).

Female reproductive tract organoids can be generated from fresh, cryopreserved biopsies and even menstrual flow samples, reflecting the donor’s physiological and genetic background (Kessler *et al.*, 2015; Kopper *et al.*, 2019; Bui *et al.*, 2020; Cindrova-Davies *et al.*, 2021; Rizo *et al.*, 2025). These models can be established from tissue representing different cycle phases, including secretory, proliferative, decidual, and atrophic endometrium. Once established, organoids grow long-term through the expansion of individual cells (Fig. 2B) and acquire a ‘conserved’ epithelial polarity (the apical or luminal surface is enclosed (apical-in), whereas the basolateral surface faces the outside and interacts with the extracellular matrix (ECM) (basolateral-out), Fig. 2C and D) (Kessler *et al.*, 2015; Boretto *et al.*, 2017; Turco *et al.*, 2017). Importantly, organoids secrete a variety of proteins, lipids, metabolites, and EVs that reflect features of reproductive tract fluid composition, making them attractive candidates for developing next-generation biomimetic culture supplements (Simintiras *et al.*, 2021; Dong *et al.*, 2023).

## Modeling oviduct and endometrial epithelial function

Despite the importance of the fallopian tube (FT), in-depth investigations have historically been constrained by limited access to primary tissue and the short lifespan of *ex vivo* epithelial cultures. Fallopian tube organoids (FTOs) overcome these constraints by allowing the formation of polarized epithelia composed of secretory (PAX8^+^) and ciliated acetylated (tubulin^+^) cells, interconnected by tight junctions. FTOs are responsive to physiological concentrations of hormones (estradiol and progesterone), inducing the expression of differentiation markers reflective of reproductive phases and exhibiting minimal transcriptional divergence from native tissue (Kessler *et al.*, 2015). FTOs are also versatile for practical utility, with the apical compartment outperforming commercial media in maintaining human sperm motility and viability for up to 96 h (Gatimel *et al.*, 2025). This reinforces FTOs as powerful platforms for studying gamete–epithelium interactions, hormone signaling, early fertilization dynamics, and disease modeling in a patient-specific context (Yucer *et al.*, 2021).

**Figure 3.**
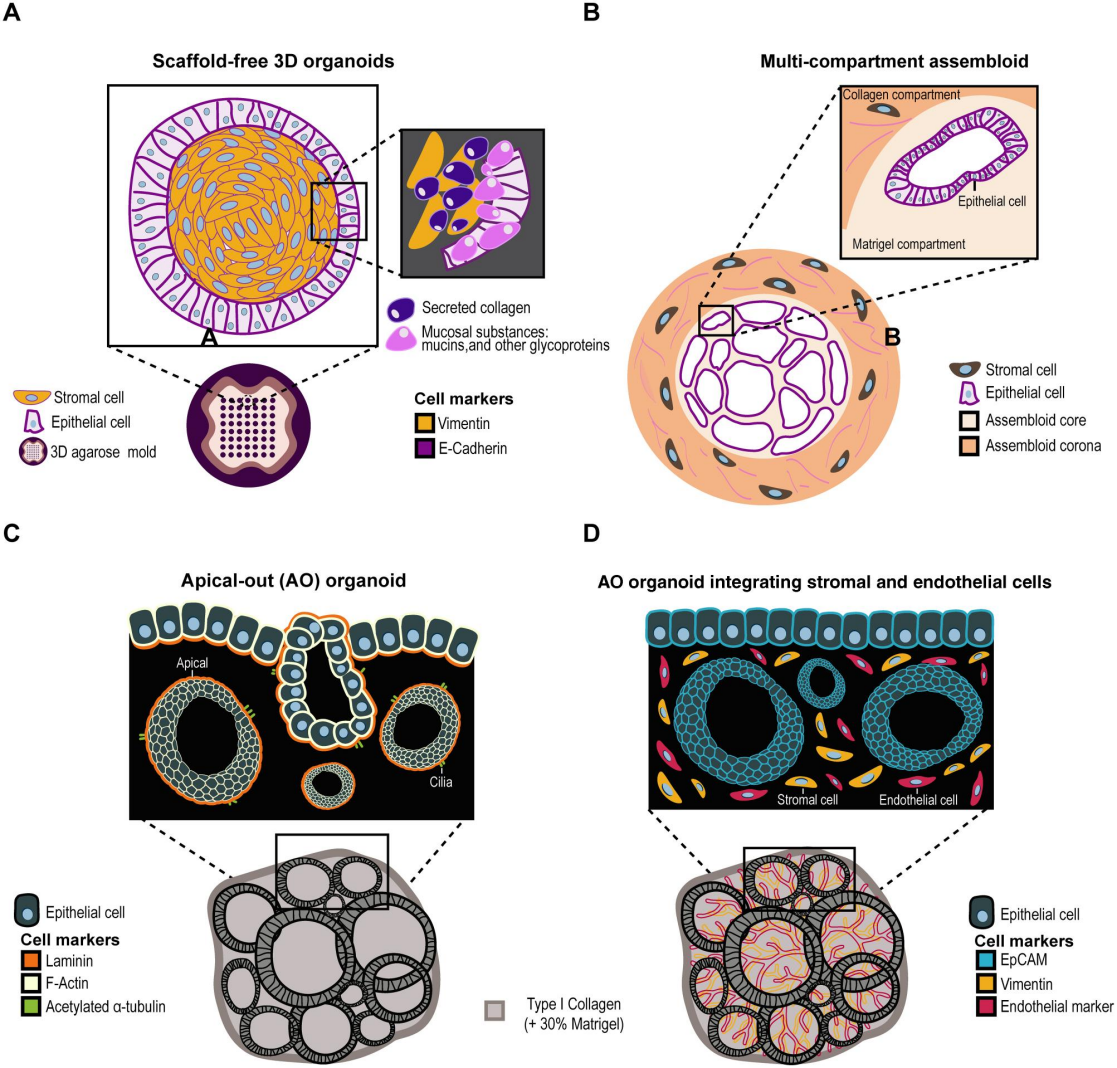
**Approaches for generating advanced endometrial organoids and multicellular assembloids.** (**A**) Scaffold-free three-dimensional organoids generated using agarose micromolds, containing both epithelial and stromal cell populations. The inset illustrates the secretion of structural and mucosal components, including collagen, mucins, and glycoproteins. Immunostaining for cell-type-specific markers, such as vimentin (stromal cells) and E-cadherin (epithelial cells), highlights the spatial organization of the different compartments. (**B**) Multicellular assembloid model comprising a central epithelial compartment surrounded by a stromal cell layer, partially recapitulating epithelial-stromal tissue architecture. (**C**) Apical-out organoid configuration in which the epithelial apical (luminal) surface is oriented outward, allowing direct exposure to the external environment. Insets show ciliated epithelial structures and luminal organization. Cytoskeletal and polarity markers, including basal lamina components, filamentous actin, and acetylated α-tubulin, illustrate epithelial polarity and cellular specialization. (**D**) Apical-out organoid incorporating epithelial, stromal, and endothelial cell populations to model epithelial–stromal–vascular interactions. Cell-type-specific markers confirm the presence and organization of the three cellular compartments. AO, apical-out.

Endometrial organoids (EMOs) emulate, at a certain level, the uterine complexity. EMOs express canonical markers of glandular epithelium (*MUC1*, *E-CADHERIN*, *CK7*, and *EPCAM*) (Gnecco *et al.*, 2023) and produce luminal/apical secretions according to hormonal stimulations. Upon exposure to estradiol, EMOs undergo proliferative expansion, upregulate key genes, such as *ESR1*, *TRH*, *MCM2–4*, and *OLFM4*, and exhibit pseudostratified glandular morphology characteristic of the *in vivo* proliferative endometrium (Boretto *et al.*, 2017; Turco *et al.*, 2017). Subsequent progesterone treatment induces a secretory phenotype, marked by glandular folding, ciliation, and increased mucin production (Fig. 3A). This is paralleled by the upregulation of genes associated with secretory differentiation and early pregnancy, including *PAEP*, *SPP1*, *MUC1*, *ALOX15*, *AQP3*, and *17βHDS2* (Boretto *et al.*, 2017; Fitzgerald *et al.*, 2023).

It is worth mentioning that the use of 3D advanced cultures aligns with the 3Rs framework for animal studies. These models can reduce the number of animals required for validation and refine experimental design by enabling controlled models of hormone response and cell communication. Additionally, because organoids retain donor-specific transcriptional and epigenetic traits, they offer opportunities to investigate individual variability in receptivity and ART outcomes, something that cannot be captured in traditional animal models.

## Bridging animal models and clinical ART: the emerging role of organoid-derived secretions

High-resolution transcriptomics, proteomics, and metabolomics analyses of human organoid-derived secretions (ODS) now enable systematic mapping of epithelial secretory pathways and direct comparisons to *in vivo* fluids across the proliferative, secretory and peri-implantation window (Dong *et al.*, 2023). ODS secreted factors not only provide nutrients, but they also modulate embryo requirements, endometrial receptivity trophoblast invasion, and immunological responses (Salamonsen *et al.*, 2013; Simintiras *et al.*, 2021).

When secretions of human EMOs generated under mid-to-late luteal phase hormone stimulation are applied to macrophages, the cells shift their phenotype toward a decidual-like profile, characterized by reduced pro-inflammatory signaling and increased markers of tissue remodeling and tolerance (e.g. CD209, NRP1) (Lin *et al.*, 2023). Extending this argument, metabolites from extraorganoid fluid (EOF) and intraorganoid fluid (IOF) of these EMOs are enriched in metabolic and immunomodulatory molecules, such as hypoxanthine, which is needed for nucleotide synthesis during rapid embryonic cell divisions, and spermine, a polyamine that modulates cytokine balance and facilitates decidualization (Simintiras *et al.*, 2021).

Currently, the application of ODS to embryo culture is no longer theoretical. Novel studies employing bovine organoid-derived, cargo-specific oviductal EVs, as well as diestrus-phase endometrial ODS (organoids hormonally programmed with estradiol and medroxyprogesterone acetate), have demonstrated their capacity to maintain/improve the quality of *in vitro*-produced embryos (Devkota *et al.*, 2026; Menjivar *et al.*, 2026). Importantly, emerging evidence further indicates that ODS derived from hormonally responsive oviductal organoids can also promote sperm capacitation (Navarro-Serna *et al.*, 2026), thereby highlighting their broader role in coordinating gamete–embryo interactions. Metabolomic profiling further supports the physiological relevance of these systems. For instance, the detection of metabolites such as isobutyrylcarnitine (normally present in native bovine UF) within bovine endometrial IOF suggests that ODS recapitulates key aspects of the luminal biochemical milieu. Functionally, although not yet equivalent to synthetic oviductal fluid controls, Day 7 blastocysts cultured solely in IOF, without the need for a standard commercial culture medium, exhibit prolonged survival, increased hatching rates, and enhanced trophectoderm specification by day 10 (Devkota *et al.*, 2026). So far, the results indicate that the intrinsic secretory programs of ODS, particularly when synchronized to relevant hormonal states, can generate a microenvironment that supports embryogenesis more effectively than conventional media.

## Strengths and current limitations of reproductive tract organoids

Overall, these findings position organoid systems as ‘secretory bioreactors’, laying the conceptual groundwork for next-generation embryo culture strategies. For this transition to occur, researchers should bear in mind that while organoids successfully reproduce epithelial signaling, they lack the stromal, vascular, immune, and endocrine cell interactions present *in vivo*. As a result, their secretomes, though informative, do not fully capture the complexity or dynamic hormonal composition of natural reproductive fluids.

The composition of ODS can also shift over time depending on passage number, media formulation, and the degree of cell differentiation. In addition, ECM scaffolds such as Matrigel (ECM derived from the Engelbreth-Holm-Swarm mouse sarcoma) introduce non-physiological components and limitations for mechanistic and translational studies. All these variables and phenotypic drifts challenge reproducibility and raise questions about whether ODS can be produced with the consistency required for clinical application. Despite the intrinsic variability of ODS, the modularity of organoids remains an attractive proof-of-concept for identifying and characterizing physiological factors under certain *in vitro* conditions. Looking ahead, ODS opens the possibility to adjust them to specific embryonic stages, physiological states (follicular versus luteal phases), or patient-specific biobanks. Achieving clinical applicability does not require eliminating biological variation; rather, it requires defining acceptable ranges and reproducible manufacturing steps, as is done for other complex biological products (e.g. human serum albumin, platelet lysates, and conditioned media used in cell therapy).

In response to some of these limitations, a scaffold-free EMO model was generated using low-adhesion agarose micromoulds to produce apical-out (AO) spheroids with an epithelial perimeter and stromal core (Fig. 3A) (Wiwatpanit *et al.*, 2020). Although originally designed to study polycystic ovary syndrome, its AO architecture provides an accessible luminal interface (Fig. 3A and C), offering a unique opportunity to directly expose gametes or embryos to native apical secretions. Similarly, AO-EMOs incorporating stromal and endothelial cells allow secretions from the internal gland-like epithelial cells to diffuse to the exterior (Fig. 3C), thereby modulating cell behavior in ways consistent with early pregnancy, as happens when they are co-cultured with human blastocysts or their *in vitro* counterparts: derived blastoids (Shibata *et al.*, 2024).

## Roadmap to target ODS in translational ART media supplements

At present, we lack standardized benchmarks to define what an “optimal” or “physiologically relevant” organoid secretome should look like. Moreover, it remains unclear whether all components of these secretions (e.g. mRNAs, signaling proteins, redox enzymes, and EV-associated factors) retain their biological activity throughout the entire IVC period or whether some undergo degradation or inactivation. Addressing these issues will require parallel phenotypic and molecular characterization of organoids (e.g. transcriptomic markers of differentiation state) alongside quantitative proteomic/EV profiling of their secretions.

Knowing which molecules are necessary and sufficient to recapitulate the beneficial effects of native reproductive fluids requires a systematic fractionation and functional testing of individual groups of molecules (proteins, lipids, metabolites, and EV-associated cargo). Following this, establishing a standard protocol and sample origin/use among laboratories, and determining the optimal concentration ranges for secreted factors, are crucial parameters that should be evaluated hereafter. Studies must also be accompanied by new quality control pipelines, including functional assays (such as embryo developmental kinetics, zona pellucida remodeling, or metabolic readouts), which are essential for determining potency rather than simply relying on the presence or absence of factors.

A practical intermediate step would be to evaluate these secretomes in well-established model systems, such as mouse or bovine, where developmental endpoints and embryo competence are routinely measured and where ethical and logistical barriers are comparatively lower. Moreover, the recent development of human blastoids and embryo-like structures (Liu *et al.*, 2021b; Kagawa *et al.*, 2022) provides an additional platform to assess how ODS influence early developmental trajectories in a controlled and ethically regulated framework (Kagawa *et al.*, 2022; Shibata *et al.*, 2024). Addressing these questions will not only improve reproducibility but also clarify how close we are to achieving a physiologically meaningful, translationally relevant *in vitro* milieu.

## Toward more complex and physiologically accurate systems: assembloids

Assembloids provide a major step forward for studying reproductive tract secretions because their multicellular organization restores epithelial-stromal communication (with unprecedented *in vitro* fidelity, Figs 2 and 3): a key driver of physiologically relevant secretome profiles, including during the window of implantation (Zhang *et al.*, 2024). Unlike epithelial-only organoids, human endometrial assembloids generate hormone-responsive secretions that more accurately represent the transition to a receptive state. Stromal cells further contribute with decidual products such as PRL, IGFBP1, and senescence-associated signals that shape the composition and timing of the essential molecules for implantation (Gnecco *et al.*, 2023; Tian *et al.*, 2023). Beyond physiological modeling, endometrial assembloids retain donor-specific signatures and capture clinically relevant alterations, such as progesterone-resistant endometriosis, adenomyosis, and recurrent implantation failure (Rawlings *et al.*, 2021; Stratopoulou *et al.*, 2025), making them highly relevant for understanding implantation failure and ART outcomes.

Current multicompartment assembloids (MA) are innovative constructs that remain in earlier stages of development. FT MA incorporates a basement membrane-like Matrigel core containing the epithelial cells, surrounded by a collagen I-rich stromal compartment (Fig. 3B), recreating the epithelial-stromal organization and mucosal folding characteristic of the *in vivo* FT. This architecture enhances epithelial differentiation, motile ciliation, and associated proteomic diversity, resulting in luminal secretions and fluid dynamics that resemble native tubal physiology. Moreover, cilia developed by the FT assembloids are not only present but functionally active, producing directional ciliary beating sufficient to transport oocyte-sized cargo along the lumen-facing epithelial surface, recapitulating a fundamental mechanical function of the FT (Crawford *et al*., 2024).

A parallel effort has focused on building endometrial MAs. This platform recapitulates the proliferative, secretory, and menstrual regression phases within a single culture system, supports decidualization, and reveals compartment-specific paracrine signaling. By uniting tissue-informed design with full-cycle hormonal responsiveness, these endometrial MA establish a powerful foundation for studying implantation, modeling gynecological disease, and advancing precision reproductive diagnostics (Ren *et al.*, 2025).

**Figure 4.**
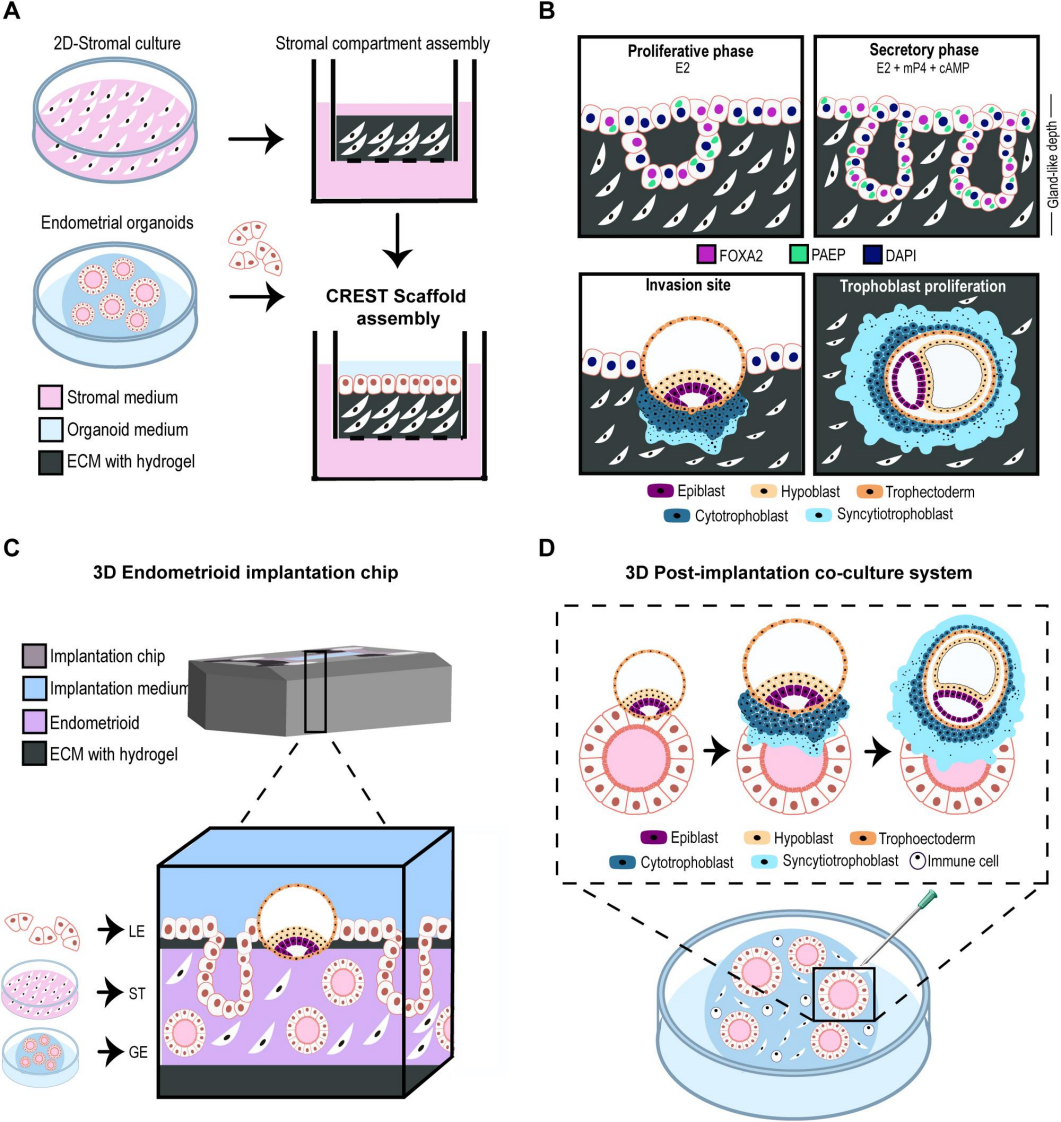
**Three-dimensional models for studying embryo implantation and early post-implantation development.** (**A**) Schematic representation of a reconstructed endometrial scaffold generated by layering a stromal hydrogel within a compartmentalized culture system and overlaying it with organoid-derived epithelial fragments to form a luminal epithelial layer. Separate culture media are used for stromal and epithelial compartments to preserve cell-specific differentiation. This engineered platform recreates stromal-epithelial architecture and supports a hormonally responsive endometrial environment. (**B**) Hormone-driven differentiation and embryo interaction within the reconstructed endometrial model. Upper panels show epithelial remodeling in response to hormonal stimulation. Under estrogen exposure, the epithelium displays a proliferative phenotype with shallow gland-like structures. Subsequent treatment with progesterone and cyclic AMP induces a secretory phenotype, characterized by increased glandular complexity and expression of differentiation markers. Lower panels illustrate embryo or blastoid attachment and invasion, including trophoblast expansion and differentiation into distinct lineages surrounding embryonic compartments. (**C**) Three-dimensional endometrial implantation model within a microfluidic platform. A hormonally primed endometrial tissue is assembled through sequential layering of extracellular matrix, stromal cells, glandular epithelial organoids, and a luminal epithelial layer. This compartmentalized system enables controlled embryo attachment and invasion under dynamic culture conditions. (**D**) Three-dimensional post-implantation co-culture model incorporating epithelial, stromal, immune, and supporting-cell populations. Organoid-derived epithelial structures are hormonally primed to mimic the receptive phase of the endometrium. Embryos are introduced into a defined niche within the structure, allowing modelling of key implantation processes, including attachment, epithelial penetration, and trophoblast differentiation, within a stabilized *in vitro* microenvironment. cAMP, cyclic adenosine monophosphate; E2, estradiol; GE, glandular epithelial organoids; LE, luminal epithelial cells; mP4, medroxyprogesterone acetate; ST, stromal cells.

Receptive endometrial scaffold platforms like the cell-engineered receptive endometrial scaffold technology (Fig. 4A and B), the 3D endometrioid on a chip (Fig. 4C), and the 3D post-implantation co-culture system (Fig. 4D) are even able to model the early human embryo and blastoid implantation process *in vitro*, recapitulating key post-implantation hallmarks such as yolk sac formation, primordial germ cell specification, and early placental development (Dong *et al.*, 2025; Molè *et al.*, 2026; Song *et al.*, 2026). Crucially, these models not only advance our understanding of maternal–embryo communication but also faithfully reproduce the luminal, glandular, and stromal compartments of the receptive endometrium (Fig. 4B), providing a physiologically relevant context for the study and collection of *in vitro* secretions, including those involved in oviductal, endometrial, and embryonic interactions (Dong *et al.*, 2025; Molè *et al.*, 2026; Song *et al.*, 2026). Moreover, these overcome the limitations of accessibility and ethical constraints in early pregnancy research. All these features position assembloids as an intermediate system between current organoid cultures, which lack complex tissue interactions, and whole-uterus models, which remain experimentally constrained.

## Next-generation models of reproductive function and diseases: vascularized assembloids

Future studies should integrate more physiologically relevant communication between epithelial, stromal, and vascular compartments into reproductive assembloids. In this way, next-generation secretomes would more faithfully reflect *in vivo* uterine and oviductal environments. Advances such as apical-out endometrial organoids incorporating stromal and endothelial components (Fig. 3C and D) provide a blueprint for engineering reproductive assembloids in which endothelial networks contribute angiocrine factors essential for receptivity, decidual remodelling, and early embryo support. Looking ahead, the incorporation of immune components may further enhance the physiological relevance of these models.

Additionally, the incorporation of micropatterned hydrogel scaffolds or hybrid microfluidic platforms with cell culture inserts could further refine secretion dynamics by recreating tissue-specific architecture, such as glandular invaginations in the endometrium or the folded ciliated surface of the fallopian tube. These features are analogous to villus-crypt-like interfaces that enhance intestinal maturation (Shin and Kim, 2022). Such patterned systems would enable spatially organized secretory activity, including hormone-mediated signaling and epithelial–stromal–vascular interactions, as well as embryo or trophoblast engagement under physiologically relevant mechanical and biochemical cues.

## Conclusions

*In vitro* embryo development, while foundational to both human ART and livestock breeding programs, continues to fall short of replicating the dynamic and finely tuned environment of the female reproductive tract. Efforts to supplement culture media with reproductive fluids have demonstrated improved embryo quality, developmental competence, and epigenetic stability. However, practical limitations, including donor variability, biosafety concerns, and lack of standardization, have restricted their widespread implementation.

ODS, 3D IVC (organoids and assembloids), scaffolds, chips, and co-culture systems now offer a scalable and ethically viable alternative. These models generate bioactive secretomes that closely mimic oviductal and UFs, providing a physiologically relevant and reproducible source of factors essential for early development. In human assisted reproductive technologies, 3D-based platforms hold the potential to personalize embryo culture conditions, reduce reliance on undefined supplements, and improve implantation and long-term developmental outcomes.

As the field advances, the integration of organoid technologies, EV biology, and functional co-culture systems represents a transformative shift toward biomimetic embryo culture. These innovations not only bridge the gap between *in vivo* and *in vitro* environments but also offer translational benefits supporting safer, more effective, and physiologically informed reproductive strategies in clinical settings.

## Data Availability

No new data were generated or analyzed in support of this article.

## References

[hoag028-B1] Adamson GD , Creighton P, de Mouzon J, Zegers-Hochschild F, Dyer S, Chambers GM. How many infants have been born with the help of assisted reproductive technology? Fertil Steril 2025;124:40–50. 39947276 10.1016/j.fertnstert.2025.02.009

[hoag028-B2] Ahlström A , Lundin K, Cimadomo D, Coticchio G, Selleskog U, Westlander G, Winerdal J, Stenfelt C, Callender S, Nyberg C et al No major differences in perinatal and maternal outcomes between uninterrupted embryo culture in time-lapse system and conventional embryo culture. Hum Reprod 2023;38:2400–2411. 37879843 10.1093/humrep/dead219

[hoag028-B3] Ahn J , Yoon M-J, Hong S-H, Cha H, Lee D, Koo HS, Ko J-E, Lee J, Oh S, Jeon NL et al Three-dimensional microengineered vascularised endometrium-on-a-chip. Hum Reprod 2021;36:2720–2731. 34363466 10.1093/humrep/deab186 PMC8450871

[hoag028-B4] Aksu DA , Agca C, Aksu S, Bagis H, Akkoc T, Caputcu AT, Arat S, Taskin AC, Kizil SH, Karasahin T et al Gene expression profiles of vitrified in vitro‐ and in vivo‐derived bovine blastocysts. Mol Reprod Dev 2012;79:613–625. 22778065 10.1002/mrd.22068

[hoag028-B5] Apostolov A , Mladenović D, Tilk K, Lõhmus A, Baev V, Yahubyan G, Sola-Leyva A, Bergamelli M, Görgens A, Zhao C et al Multi-omics analysis of uterine fluid extracellular vesicles reveals a resemblance with endometrial tissue across the menstrual cycle: biological and translational insights. Hum Reprod Open 2025;2025:hoaf010. 40084293 10.1093/hropen/hoaf010 PMC11904304

[hoag028-B6] Auvinen P , Vehviläinen J, Rämö K, Laukkanen I, Marjonen-Lindblad H, Wallén E, Söderström-Anttila V, Kahila H, Hydén-Granskog C, Tuuri T et al Genome-wide DNA methylation and gene expression in human placentas derived from assisted reproductive technology. Commun Med (Lond) 2024;4:267. 39702541 10.1038/s43856-024-00694-6 PMC11659305

[hoag028-B7] Baker VL , Dyer S, Chambers GM, Keller E, Banker M, de Mouzon J, Elgindy E, Bai FM, Ishihara O, Jwa SC et al International Committee for Monitoring Assisted Reproductive Technologies (ICMART): world report for cycles conducted in 2017–2018. Hum Reprod 2025;40:1110–1126. 40239109 10.1093/humrep/deaf049

[hoag028-B8] Barberet J , Ducreux B, Guilleman M, Simon E, Bruno C, Fauque P. DNA methylation profiles after ART during human lifespan: a systematic review and meta-analysis. Hum Reprod Update 2022;28:629–655. 35259267 10.1093/humupd/dmac010

[hoag028-B9] Benedetti C , Pavani KC, Gansemans Y, Azari-Dolatabad N, Pascottini OB, Peelman L, Six R, Fan Y, Guan X, Deserranno K et al From follicle to blastocyst: microRNA-34c from follicular fluid-derived extracellular vesicles modulates blastocyst quality. J Anim Sci Biotechnol 2024;15:104. 39097731 10.1186/s40104-024-01059-8 PMC11298084

[hoag028-B10] Bongso A , Soon-Chye N, Sathananthan H, Lian NP, Rauff M, Ratnam S. Improved quality of human embryos when co-cultured with human ampullary cells. Hum Reprod 1989;4:706–713. 2778057 10.1093/oxfordjournals.humrep.a136971

[hoag028-B11] Boretto M , Cox B, Noben M, Hendriks N, Fassbender A, Roose H, Amant F, Timmerman D, Tomassetti C, Vanhie A et al Development of organoids from mouse and human endometrium showing endometrial epithelium physiology and long-term expandability. Development 2017;144:1775–1786. 28442471 10.1242/dev.148478

[hoag028-B563881] Borland RM, , Biggers JD, , Lechene CP, , Taymor ML. Elemental composition of fluid in the human Fallopian tube. J Reprod Fertil 1980;58:479–482. 7431281 10.1530/jrf.0.0580479

[hoag028-B12] Bourdon G , Cadoret V, Charpigny G, Couturier-Tarrade A, Dalbies-Tran R, Flores M-J, Froment P, Raliou M, Reynaud K, Saint-Dizier M et al Progress and challenges in developing organoids in farm animal species for the study of reproduction and their applications to reproductive biotechnologies. Vet Res 2021;52:42. 33691745 10.1186/s13567-020-00891-w PMC7944619

[hoag028-B13] Bragança GM , Alcântara-Neto AS, Batista RITP, Brandão FZ, Freitas VJF, Mermillod P, Souza-Fabjan JMG. Oviduct fluid during IVF moderately modulates polyspermy in in vitro-produced goat embryos during the non-breeding season. Theriogenology 2021;168:59–65. 33857909 10.1016/j.theriogenology.2021.03.022

[hoag028-B14] Bui BN , Boretto M, Kobayashi H, van Hoesel M, Steba GS, van Hoogenhuijze N, Broekmans FJM, Vankelecom H, Torrance HL. Organoids can be established reliably from cryopreserved biopsy catheter-derived endometrial tissue of infertile women. Reprod Biomed Online 2020;41:465–473. 32622705 10.1016/j.rbmo.2020.03.019

[hoag028-B15] Busch C , Hill CJ, Paterson K, Mellin R, Zagnoni M, Hapangama DK, Sandison ME. Functional, patient-derived 3D tri-culture models of the uterine wall in a microfluidic array. Hum Reprod 2024;39:2537–2550. 39277544 10.1093/humrep/deae214 PMC11532614

[hoag028-B16] Consensus Group C. ‘There is only one thing that is truly important in an IVF laboratory: everything’ Cairo Consensus Guidelines on IVF Culture Conditions. Reprod Biomed Online 2020;40:33–60. 31836437 10.1016/j.rbmo.2019.10.003

[hoag028-B17] Canha-Gouveia A , Prieto-Sánchez MT, Sánchez-Ferrer ML, Mollá M, Martínez-Soto JC, París-Oller E, Soriano-Úbeda C, Landeras J, Coy P. Physicochemical and functional characterization of female reproductive fluids: a report of the first two infants born following addition of their mother’s fluids to the embryo culture media. Front Physiol 2021;12:710887. 34552502 10.3389/fphys.2021.710887 PMC8451538

[hoag028-B18] Canovas S , Ivanova E, Romar R, García-Martínez S, Soriano-Úbeda C, García-Vázquez FA, Saadeh H, Andrews S, Kelsey G, Coy P. DNA methylation and gene expression changes derived from assisted reproductive technologies can be decreased by reproductive fluids. eLife 2017;6:e23670. 28134613 10.7554/eLife.23670 PMC5340525

[hoag028-B5921835378] Casslén B, , Nilsson B. Human uterine fluid, examined in undiluted samples for osmolarity and the concentrations of inorganic ions, albumin, glucose, and urea. Am J Obstet Gynecol 1984;150:877–881. 6507514 10.1016/0002-9378(84)90466-6

[hoag028-B19] Chronopoulou E , Harper JC. IVF culture media: past, present and future. Hum Reprod Update 2015;21:39–55. 25035437 10.1093/humupd/dmu040

[hoag028-B20] Cindrova-Davies T , Zhao X, Elder K, Jones CJP, Moffett A, Burton GJ, Turco MY. Menstrual flow as a non-invasive source of endometrial organoids. Commun Biol 2021;4:651. 34140633 10.1038/s42003-021-02194-y PMC8211845

[hoag028-B21] Costa-Borges N , Munné S, Albó E, Mas S, Castelló C, Giralt G, Lu Z, Chau C, Acacio M, Mestres E et al First babies conceived with Automated Intracytoplasmic Sperm Injection. Reprod Biomed Online 2023;47:103237. 37400320 10.1016/j.rbmo.2023.05.009

[hoag028-B22] Coy P , Cánovas S, Mondéjar I, Saavedra MD, Romar R, Grullón L, Matás C, Avilés M. Oviduct-specific glycoprotein and heparin modulate sperm–zona pellucida interaction during fertilization and contribute to the control of polyspermy. Proc Natl Acad Sci USA 2008;105:15809–15814. 18838686 10.1073/pnas.0804422105 PMC2572915

[hoag028-B23] Coy P , García-Vázquez FA, Visconti PE, Avilés M. Roles of the oviduct in mammalian fertilization. Reproduction 2012;144:649–660. 23028122 10.1530/REP-12-0279 PMC4022750

[hoag028-B229692194] Crawford AJ, , Forjaz A, , Bons J, , Bhorkar I, , Roy T, , Schell D, , Queiroga V, , Ren K, , Kramer D, , Huang W et al Combined assembloid modeling and 3D whole-organ mapping captures the microanatomy and function of the human fallopian tube. Sci Adv 2024;10:eadp6285. 39331707 10.1126/sciadv.adp6285 PMC11430475

[hoag028-B24] Cui L , Zhou W, Xi B, Ma J, Hu J, Fang M, Hu K, Qin Y, You L, Cao Y et al Increased risk of metabolic dysfunction in children conceived by assisted reproductive technology. Diabetologia 2020;63:2150–2157. 32757153 10.1007/s00125-020-05241-1

[hoag028-B25] Devkota I , Bonomo ZL, Fuego DM, Li Y, Zhang X, Loux SC, Looney CR, Maia AIV, Donnarumma F, Matsakas A et al Establishment and functional characterization of bovine endometrial epithelial organoids. FASEB J 2026;40:e71515. 41653001 10.1096/fj.202503351R

[hoag028-B26] Dong L , Sun X, An S, Xiang J, Hu L, Yao D, Chang J, Jia R, Yang Y, Wang S. Mechanistic insights into recurrent implantation failure: the lactate-H3K18la-SLC7A11 axis explored via endometrial organoid and blastoid–endometrial cell implantation models. Cell Prolif 2025;16:e70147. 10.1111/cpr.70147 PMC13241822 41242872

[hoag028-B27] Dong Y , Li J, Cao D, Zhong J, Liu X, Duan Y-G, Lee K-F, Yeung WSB, Lee C-L, Chiu PCN. Integrated microRNA and secretome analysis of human endometrial organoids reveal the miR-3194-5p/aquaporin/S100A9 module in regulating trophoblast functions. Mol Cell Proteomics 2023;22:100526. 36889440 10.1016/j.mcpro.2023.100526 PMC10119685

[hoag028-B28] Driver AM , Peñagaricano F, Huang W, Ahmad KR, Hackbart KS, Wiltbank MC, Khatib H. RNA-Seq analysis uncovers transcriptomic variations between morphologically similar in vivo- and in vitro-derived bovine blastocysts. BMC Genomics 2012;13:118. 22452724 10.1186/1471-2164-13-118 PMC3368723

[hoag028-B29] Dumoulin JC , Land JA, Van Montfoort AP, Nelissen EC, Coonen E, Derhaag JG, Schreurs IL, Dunselman GA, Kester AD, Geraedts JP et al Effect of in vitro culture of human embryos on birthweight of newborns. Hum Reprod 2010;25:605–612. 20085915 10.1093/humrep/dep456

[hoag028-B30] Edwards RG , Bavister BD, Steptoe PC. Early stages of fertilization in vitro of human oocytes matured in vitro. Nature 1969;221:632–635. 4886881 10.1038/221632a0

[hoag028-B31] Edwards RG , Steptoe PC, Purdy JM. Fertilization and cleavage in vitro of preovulator human oocytes. Nature 1970;227:1307–1309. 4916973 10.1038/2271307a0

[hoag028-B32] Ferraz MAMM , Rho HS, Hemerich D, Henning HHW, van Tol HTA, Hölker M, Besenfelder U, Mokry M, Vos PLAM, Stout TAE et al An oviduct-on-a-chip provides an enhanced in vitro environment for zygote genome reprogramming. Nat Commun 2018;9:4934. 30467383 10.1038/s41467-018-07119-8 PMC6250703

[hoag028-B33] Fitzgerald HC , Kelleher AM, Ranjit C, Schust DJ, Spencer TE. Basolateral secretions of human endometrial epithelial organoids impact stromal cell decidualization. Mol Hum Reprod 2023;29:gaad007. 36821428 10.1093/molehr/gaad007 PMC10321591

[hoag028-B34] Flaquer A , Baumbach C, Kriebel J, Meitinger T, Peters A, Waldenberger M, Grallert H, Strauch K. Mitochondrial genetic variants identified to be associated with BMI in adults. PLoS One 2014;9:e105116. 25153900 10.1371/journal.pone.0105116 PMC4143221

[hoag028-B35] Gatimel N , Perez G, Bruno E, Sagnat D, Rolland C, Tanguy-Le-Gac Y, Di Donato E, Racaud C, Léandri R, Bettiol C et al Human fallopian tube organoids provide a favourable environment for sperm motility. Hum Reprod 2025;40:503–517. 39792911 10.1093/humrep/deae258

[hoag028-B36] Gnecco JS , Brown A, Buttrey K, Ives C, Goods BA, Baugh L, Hernandez-Gordillo V, Loring M, Isaacson KB, Griffith LG. Organoid co-culture model of the human endometrium in a fully synthetic extracellular matrix enables the study of epithelial-stromal crosstalk. Med 2023;4:554–579.e9. 37572651 10.1016/j.medj.2023.07.004 PMC10878405

[hoag028-B37] Gnecco JS , Pensabene V, Li DJ, Ding T, Hui EE, Bruner-Tran KL, Osteen KG. Compartmentalized culture of perivascular stroma and endothelial cells in a microfluidic model of the human endometrium. Ann Biomed Eng 2017;45:1758–1769. 28108942 10.1007/s10439-017-1797-5 PMC5489603

[hoag028-B38] Gnoth C , Maxrath B, Skonieczny T, Friol K, Godehardt E, Tigges J. Final ART success rates: a 10 years survey. Hum Reprod 2011;26:2239–2246. 21659314 10.1093/humrep/der178

[hoag028-B39] Godakumara K , Heath PR, Fazeli A. Rhythm of the first language: dynamics of extracellular vesicle-based embryo–maternal communication in the pre-implantation microenvironment. Int J Mol Sci 2023;24:6811. 37047784 10.3390/ijms24076811 PMC10095160

[hoag028-B40] Gonella-Diaza A , Sponchiado M, Rodrigues França M, Liu L, Pugliesi G, Guimarães Lo Turco E, Peñagaricano F, Binelli M. The metabolomic composition of the oviductal fluid is controlled by the periovulatory hormonal context in *Bos indicus* cows. Biol Reprod 2024;111:1188–1201. 39418319 10.1093/biolre/ioae153

[hoag028-B41] Håberg SE , Page CM, Lee Y, Nustad HE, Magnus MC, Haftorn KL, Carlsen EØ, Denault WRP, Bohlin J, Jugessur A et al DNA methylation in newborns conceived by assisted reproductive technology. Nat Commun 2022;13:1896. 35393427 10.1038/s41467-022-29540-w PMC8989983

[hoag028-B42] Hamdi M , Lopera-Vasquez R, Maillo V, Sanchez-Calabuig MJ, Núnez C, Gutierrez-Adan A, Rizos D. Bovine oviductal and uterine fluid support in vitro embryo development. Reprod Fertil Dev 2018;30:935–945. 29167013 10.1071/RD17286

[hoag028-B43] Hamdi M , Sánchez JM, Fernandez-Fuertes B, Câmara DR, Bollwein H, Rizos D, Bauersachs S, Almiñana C. Oviductal extracellular vesicles miRNA cargo varies in response to embryos and their quality. BMC Genomics 2024;25:520. 38802796 10.1186/s12864-024-10429-5 PMC11129498

[hoag028-B44] Heras S , Lopes JS, Quintero-Moreno A, Romero-Aguirregomezcorta J, Canovas S, Romar R, Coy P. Growth parameters and growth-related hormone profile in a herd of cattle up to 4 years of age derived from assisted reproductive technologies. Animals 2025a;15:631. 40075913 10.3390/ani15050631 PMC11898124

[hoag028-B45] Heras S , Soriano-Ubeda C, Quintero-Moreno A, Romero-Aguirregomezcorta J, Paris-Oller E, Gadea J, Romar R, Canovas S, Coy P. Growth performance in pigs derived from in vitro produced embryos is enhanced compared to their artificial insemination-derived counterparts from birth to adulthood. Theriogenology 2025b;239:117372. 40058118 10.1016/j.theriogenology.2025.117372

[hoag028-B46] Kagawa H , Javali A, Khoei HH, Sommer TM, Sestini G, Novatchkova M, Scholte Op Reimer Y, Castel G, Bruneau A, Maenhoudt N et al Human blastoids model blastocyst development and implantation. Nature 2022;601:600–605. 34856602 10.1038/s41586-021-04267-8 PMC8791832

[hoag028-B47] Kessler M , Hoffmann K, Brinkmann V, Thieck O, Jackisch S, Toelle B, Berger H, Mollenkopf H-J, Mangler M, Sehouli J et al The Notch and Wnt pathways regulate stemness and differentiation in human fallopian tube organoids. Nat Commun 2015;6:8989. 26643275 10.1038/ncomms9989 PMC4686873

[hoag028-B48] Kleijkers SHM , van Montfoort APA, Smits LJM, Viechtbauer W, Roseboom TJ, Nelissen ECM, Coonen E, Derhaag JG, Bastings L, Schreurs IEL et al IVF culture medium affects post-natal weight in humans during the first 2 years of life. Hum Reprod 2014;29:661–669. 24549211 10.1093/humrep/deu025

[hoag028-B49] Koeck RM , Busato F, Tost J, Consten D, van Echten-Arends J, Mastenbroek S, Wurth Y, Remy S, Langie S, Nawrot TS et al Methylome-wide analysis of IVF neonates that underwent embryo culture in different media revealed no significant differences. NPJ Genom Med 2022;7:39. 35768464 10.1038/s41525-022-00310-3 PMC9243125

[hoag028-B50] Kölle S , Dubielzig S, Reese S, Wehrend A, König P, Kummer W. Ciliary transport, gamete interaction, and effects of the early embryo in the oviduct: ex vivo analyses using a new digital videomicroscopic system in the cow. Biol Reprod 2009;81:267–274. 19299315 10.1095/biolreprod.108.073874

[hoag028-B51] Kopper O , de Witte CJ, Lõhmussaar K, Valle-Inclan JE, Hami N, Kester L, Balgobind AV, Korving J, Proost N, Begthel H et al An organoid platform for ovarian cancer captures intra- and interpatient heterogeneity. Nat Med 2019;25:838–849. 31011202 10.1038/s41591-019-0422-6

[hoag028-B52] Leal CLV , Cañón-Beltrán K, Cajas YN, Hamdi M, Yaryes A, Millán de la Blanca MG, Beltrán-Breña P, Mazzarella R, da Silveira JC, Gutiérrez-Adán A et al Extracellular vesicles from oviductal and uterine fluids supplementation in sequential in vitro culture improves bovine embryo quality. J Anim Sci Biotechnol 2022;13:116. 36280872 10.1186/s40104-022-00763-7 PMC9594899

[hoag028-B53] Lee G , Lee Y-G, Koo HS, Hwang S-Y, Lee D, Lee J, Yu WJ, Kim YY, Ku S-Y, Park J-C et al Microengineered patient-derived endometrium-on-a-chip for the evaluation of endometrial receptivity and personalised translational medicine. Nat Commun 2025;16:10439. 41290570 10.1038/s41467-025-65406-7 PMC12647634

[hoag028-B54] Lee SH , Liu X, Jimenez-Morales D, Rinaudo PF. Murine blastocysts generated by in vitro fertilization show increased Warburg metabolism and altered lactate production. eLife 2022;11:e79153. 36107481 10.7554/eLife.79153 PMC9519152

[hoag028-B55] Li M , Zhao Z, Tao Q, Huang J, Lian Y, Li Y, Lin S, Liu P, Li Q, Li R et al Effects of different embryo culture media on birthweight following assisted reproductive technology. Hum Reprod Open 2025;2025:hoaf041. 40718349 10.1093/hropen/hoaf041 PMC12296354

[hoag028-B56] Li Y , Liu C, Guo N, Cai L, Wang M, Zhu L, Li F, Jin L, Sui C. Extracellular vesicles from human fallopian tubal fluid benefit embryo development *in vitro*. Hum Reprod Open 2023;2023:hoad006. 36895886 10.1093/hropen/hoad006 PMC9991590

[hoag028-B57] Lin L , Bai K, Li J, Chiu PCN, Lee C-L. Regulatory role of human endometrial gland secretome on macrophage differentiation. J Reprod Immunol 2023;160:104158. 37801890 10.1016/j.jri.2023.104158

[hoag028-B1087249] Lippes J, , Enders RG, , Pragay DA, , Bartholomew WR. The collection and analysis of human fallopian tubal fluid. Contraception 1972;5:85–103. 4631126 10.1016/0010-7824(72)90021-2

[hoag028-B58] Liu C , Wang M, Zhang H, Sui C. Altered microRNA profiles of extracellular vesicles secreted by endometrial cells from women with recurrent implantation failure. Reprod Sci 2021a;28:1945–1955. 33432533 10.1007/s43032-020-00440-y

[hoag028-B59] Liu C , Yao W, Yao J, Li L, Yang L, Zhang H, Sui C. Endometrial extracellular vesicles from women with recurrent implantation failure attenuate the growth and invasion of embryos. Fertil Steril 2020;114:416–425. 32622655 10.1016/j.fertnstert.2020.04.005

[hoag028-B60] Liu X , Tan JP, Schröder J, Aberkane A, Ouyang JF, Mohenska M, Lim SM, Sun YBY, Chen J, Sun G et al Modelling human blastocysts by reprogramming fibroblasts into iBlastoids. Nature 2021b;591:627–632. 33731926 10.1038/s41586-021-03372-y

[hoag028-B61] Londero AP , Orsaria M, Parisi N, Tassi A, Pittini C, Driul L, Mariuzzi L. In vitro fertilization is associated with placental accelerated villous maturation. Int J Clin Exp Pathol 2021;14:734–740. 34239675 PMC8255202

[hoag028-B62] Lopes JS , Alcázar-Triviño E, Soriano-Úbeda C, Hamdi M, Cánovas S, Rizos D, Coy P. Reproductive outcomes and endocrine profile in artificially inseminated versus embryo transferred cows. Animals 2020;10:1359. 32781545 10.3390/ani10081359 PMC7459650

[hoag028-B63] Ma Q , Beal JR, Bhurke A, Kannan A, Yu J, Taylor RN, Bagchi IC, Bagchi MK. Extracellular vesicles secreted by human uterine stromal cells regulate decidualization, angiogenesis, and trophoblast differentiation. Proc Natl Acad Sci USA 2022;119:e2200252119. 36095212 10.1073/pnas.2200252119 PMC9499590

[hoag028-B64] Mahe MM , Aihara E, Schumacher MA, Zavros Y, Montrose MH, Helmrath MA, Sato T, Shroyer NF. Establishment of gastrointestinal epithelial organoids. Curr Protoc Mouse Biol 2013;3:217–240. 25105065 10.1002/9780470942390.mo130179 PMC4120977

[hoag028-B65] Matsuzaki S , Nagase Y, Takiuchi T, Kakigano A, Mimura K, Lee M, Matsuzaki S, Ueda Y, Tomimatsu T, Endo M et al Antenatal diagnosis of placenta accreta spectrum after in vitro fertilization-embryo transfer: a systematic review and meta-analysis. Sci Rep 2021;11:9205. 33911134 10.1038/s41598-021-88551-7 PMC8080594

[hoag028-B66] Mendizabal-Ruiz G , Chavez-Badiola A, Hernández-Morales E, Valencia-Murillo R, Ocegueda-Hernández V, Costa-Borges N, Mestres E, Acacio M, Matia-Algué Q, Farías AF-S et al A digitally controlled, remotely operated ICSI system: case report of the first live birth. Reprod Biomed Online 2025;50:104943. 40210512 10.1016/j.rbmo.2025.104943

[hoag028-B67] Menjivar N , Gad A, Thompson R, Meyers M, Ghosh S, Hollinshead F, Tesfaye D. Organoids simulating the bovine oviduct mediate the embryo-maternal interface via extracellular vesicle-transmitted signaling. Hum Reprod Open 2026;2026:hoaf076. 41503164 10.1093/hropen/hoaf076 PMC12774516

[hoag028-B68] Mertens J , Belva F, van Montfoort APA, Regin M, Zambelli F, Seneca S, Couvreu de Deckersberg E, Bonduelle M, Tournaye H, Stouffs K et al Children born after assisted reproduction more commonly carry a mitochondrial genotype associating with low birthweight. Nat Commun 2024;15:1232. 38336715 10.1038/s41467-024-45446-1 PMC10858059

[hoag028-B69] Molè MA , Elderkin S, Zorzan I, Penfold C, Horsley N, Pokhilko A, Polanek M, Palomar A, Sinha M, Wang Y et al Modeling human embryo implantation in vitro. Cell 2026;189:87–105.e28. 41443191 10.1016/j.cell.2025.10.027

[hoag028-B70] Morbeck DE , Baumann NA, Oglesbee D. Composition of single-step media used for human embryo culture. Fertil Steril 2017;107:1055–1060.e1. 28238490 10.1016/j.fertnstert.2017.01.007

[hoag028-B71] Muraoka A , Yokoi A, Yoshida K, Kitagawa M, Asano-Inami E, Murakami M, Miyake N, Nakanishi N, Nakamura T, Osuka S et al Small extracellular vesicles in follicular fluids for predicting reproductive outcomes in assisted reproductive technology. Commun Med (Lond) 2024;4:33. 38418565 10.1038/s43856-024-00460-8 PMC10902298

[hoag028-B72] Navarro-Serna S , Romero-Aguirregomezcorta J, Hernández-3 Díaz N, Ferrero-Micó A, Coy P, Pérez-García V. Hormonally responsive bovine oviductal organoids recapitulate native oviductal secretions and enhance sperm capacitation. bioRxiv. doi: 10.64898/2026.03.10.710777, 2026, preprint: not peer reviewed. PMC13190940 42009932

[hoag028-B73] Nelissen ECM , Dumoulin JCM, Daunay A, Evers JLH, Tost J, van Montfoort APA. Placentas from pregnancies conceived by IVF/ICSI have a reduced DNA methylation level at the H19 and MEST differentially methylated regions†. Hum Reprod 2013;28:1117–1126. 23343754 10.1093/humrep/des459

[hoag028-B74] Pakniyat Z , Azari M, Kafi M, Ghaemi M, Hashemipour SMA, Safaie A, Eshghi D. The effect of follicular and ampullary fluid extracellular vesicles on bovine oocyte competence and in vitro fertilization rates. PLoS One 2025;20:e0325268. 40478808 10.1371/journal.pone.0325268 PMC12143572

[hoag028-B75] París-Oller E , Navarro-Serna S, Soriano-Úbeda C, Lopes JS, Matás C, Ruiz S, Latorre R, López-Albors O, Romar R, Cánovas S et al Reproductive fluids, used for the in vitro production of pig embryos, result in healthy offspring and avoid aberrant placental expression of PEG3 and LUM. J Anim Sci Biotechnol 2021;12:32. 33583428 10.1186/s40104-020-00544-0 PMC7883450

[hoag028-B76] París-Oller E , Soriano-Úbeda C, Belda-Pérez R, Sarriás-Gil L, Lopes JS, Canha-Gouveia A, Gadea J, Vieira LA, García-Vázquez FA, Romar R et al Reproductive fluids, added to the culture media, contribute to minimizing phenotypical differences between in vitro-derived and artificial insemination-derived piglets. J Dev Orig Health Dis 2022;13:593–605. 34986913 10.1017/S2040174421000702

[hoag028-B77] Párraga-Ros E , Álvarez-Martín Ú, Seva J, Coy P, Romar R. The impact of in vitro embryo production on placental and umbilical cord vascularization is minimized by the addition of reproductive fluids. Theriogenology 2023;208:149–157. 37329589 10.1016/j.theriogenology.2023.05.029

[hoag028-B78] Pavani KC , Meese T, Pascottini OB, Guan X, Lin X, Peelman L, Hamacher J, Van Nieuwerburgh F, Deforce D, Boel A et al Hatching is modulated by microRNA-378a-3p derived from extracellular vesicles secreted by blastocysts. Proc Natl Acad Sci USA 2022;119:e2122708119. 10.1073/pnas.2122708119 PMC8944274 35298333

[hoag028-B79] Pavani KC , XueFeng G, Chunduru J, Meese T, Peelman LJ, Van Nieuwerburgh F, Deforce D, Hendrix A, Tilleman K, Van Soom A, et al MicroRNA-146b negatively affects bovine embryo development and quality. Reproduction 2024;167:e230155. 38063339 10.1530/REP-23-0155

[hoag028-B80] Pavličev M , Wagner GP, Chavan AR, Owens K, Maziarz J, Dunn-Fletcher C, Kallapur SG, Muglia L, Jones H. Single-cell transcriptomics of the human placenta: inferring the cell communication network of the maternal-fetal interface. Genome Res 2017;27:349–361. 28174237 10.1101/gr.207597.116 PMC5340963

[hoag028-B81] Piibor J , Waldmann A, Dissanayake K, Andronowska A, Ivask M, Prasadani M, Kavak A, Kodithuwakku S, Fazeli A. Uterine fluid extracellular vesicles proteome is altered during the estrous cycle. Mol Cell Proteomics 2023;22:100642. 37678639 10.1016/j.mcpro.2023.100642 PMC10641272

[hoag028-B82] Pinborg A , Wennerholm U-B, Bergh C. Long-term outcomes for children conceived by assisted reproductive technology. Fertil Steril 2023;120:449–456. 37086833 10.1016/j.fertnstert.2023.04.022

[hoag028-B83] Piromlertamorn W , Saeng-Anan U, Vutyavanich T. Effects of ovarian endometriotic fluid exposure on fertilization rate of mouse oocytes and subsequent embryo development. Reprod Biol Endocrinol 2013;11:4. 23332096 10.1186/1477-7827-11-4 PMC3551806

[hoag028-B84] Qin J-B , Sheng X-Q, Wu D, Gao S-Y, You Y-P, Yang T-B, Wang H. Worldwide prevalence of adverse pregnancy outcomes among singleton pregnancies after in vitro fertilization/intracytoplasmic sperm injection: a systematic review and meta-analysis. Arch Gynecol Obstet 2017;295:285–301. 27896474 10.1007/s00404-016-4250-3

[hoag028-B85] Racowsky C , Cohen J, Gardner DK, Sakkas D, Rienzi L. Rethinking embryology dogma. Fertil Steril 2026;125:2–12. 41202946 10.1016/j.fertnstert.2025.10.030

[hoag028-B86] Rawlings TM , Makwana K, Taylor DM, Molè MA, Fishwick KJ, Tryfonos M, Odendaal J, Hawkes A, Zernicka-Goetz M, Hartshorne GM et al Modelling the impact of decidual senescence on embryo implantation in human endometrial assembloids. eLife 2021;10:e69603. 10.7554/eLife.69603 PMC8523170 34487490

[hoag028-B87] Ren KA , Duarte-Alvarado V, Di Zhou X, Ho B, Forjaz A, Crawford AJ, Alicea-Rebecca GM, Joshi S, Nair P, Hanna EA et al 3D map-guided modeling of functional endometrial tissue using multi-compartment assembloids. bioRxiv. doi: 10.1101/2025.08.22.671545, 2025, preprint: not peer reviewed.

[hoag028-B88] Rizo JA , Ahmad V, Pru JM, Winuthayanon S, Challa S, Kim TH, Jeong J-W, Spencer TE, Kelleher AM. Uterine organoids reveal insights into epithelial specification and plasticity in development and disease. Proc Natl Acad Sci USA 2025;122:e2422694122. 10.1073/pnas.2422694122 PMC11804710 39883834

[hoag028-B89] Salamonsen LA , Edgell T, Rombauts LJF, Stephens AN, Robertson DM, Rainczuk A, Nie G, Hannan NJ. Proteomics of the human endometrium and uterine fluid: a pathway to biomarker discovery. Fertil Steril 2013;99:1086–1092. 23043689 10.1016/j.fertnstert.2012.09.013

[hoag028-B90] Sato T , Stange DE, Ferrante M, Vries RGJ, van Es JH, van den Brink S, van Houdt WJ, Pronk A, van Gorp J, Siersema PD et al Long-term expansion of epithelial organoids from human colon, adenoma, adenocarcinoma, and Barrett’s epithelium. Gastroenterology 2011;141:1762–1772. 21889923 10.1053/j.gastro.2011.07.050

[hoag028-B5892194] Sato T, , Vries RG, , Snippert HJ, , Van De Wetering M, , Barker N, , Stange DE, , Van Es JH, , Abo A, , Kujala P, , Peters PJ et al Single Lgr5 stem cells build crypt-villus structures in vitro without a mesenchymal niche. Nature 2009;459:262–265. 19329995 10.1038/nature07935

[hoag028-B91] Sciorio R , Rinaudo P. Culture conditions in the IVF laboratory: state of the ART and possible new directions. J Assist Reprod Genet 2023;40:2591–2607. 37725178 10.1007/s10815-023-02934-5 PMC10643723

[hoag028-B92] Serrano-Albal M , Romero-Aguirregomezcorta J, Cánovas S, Heras S, Gadea J, Coy P, Romar R. Long-term study of physical, haematological, and biochemical parameters in cattle with different embryo origins. Animals 2025;15:1763. 40564315 10.3390/ani15121763 PMC12189840

[hoag028-B93] Shibata S , Endo S, Nagai LAE, Kobayashi H, Oike E, Kobayashi A, Kitamura N, Hori A, Nashimoto T, Nakato Y et al Modeling embryo-endometrial interface recapitulating human embryo implantation. Sci Adv 2024;10:eadi4819. 38394208 10.1126/sciadv.adi4819 PMC10889356

[hoag028-B94] Shin W , Kim HJ. 3D in vitro morphogenesis of human intestinal epithelium in a gut-on-a-chip or a hybrid chip with a cell culture insert. Nat Protoc 2022;17:910–939. 35110737 10.1038/s41596-021-00674-3 PMC9675318

[hoag028-B95] Simintiras CA , Dhakal P, Ranjit C, Fitzgerald HC, Balboula AZ, Spencer TE. Capture and metabolomic analysis of the human endometrial epithelial organoid secretome. Proc Natl Acad Sci USA 2021;118:e2026804118. 10.1073/pnas.2026804118 PMC8053979 33876774

[hoag028-B96] Song J , Zhao R, Zhang Y, Lu M, Liu P, Li T, Li C, Yu R, Chen X, Yang H et al 3D post-implantation co-culture of human embryo and endometrium. Cell Stem Cell 2026;33:58–72.e7. 41443195 10.1016/j.stem.2025.12.002

[hoag028-B97] Sonigo C , Ahdad-Yata N, Pirtea P, Solignac C, Grynberg M, Sermondade N. Do IVF culture conditions have an impact on neonatal outcomes? A systematic review and meta-analysis. J Assist Reprod Genet 2024;41:563–580. 38246922 10.1007/s10815-024-03020-0 PMC10957805

[hoag028-B98] Stratopoulou CA , Rossi M, Beaussart C, Zipponi M, Camboni A, Donnez J, Dolmans M-M. Generation of epithelial-stromal assembloids as an advanced in vitro model of impaired adenomyosis-related endometrial receptivity. Fertil Steril 2025;123:350–360. 39197515 10.1016/j.fertnstert.2024.08.339

[hoag028-B99] Sunde A , Brison D, Dumoulin J, Harper J, Lundin K, Magli MC, Van den Abbeel E, Veiga A. Time to take human embryo culture seriously: table I. Hum Reprod 2016;31:2174–2182. 27554442 10.1093/humrep/dew157

[hoag028-B100] Tarahomi M , Vaz FM, van Straalen JP, Schrauwen FAP, van Wely M, Hamer G, Repping S, Mastenbroek S. The composition of human preimplantation embryo culture media and their stability during storage and culture. Hum Reprod 2019;34:1450–1461. 31348827 10.1093/humrep/dez102

[hoag028-B101] Thompson RE , Meyers MA, Premanandan C, Hollinshead FK. Generation and cryopreservation of feline oviductal organoids. Theriogenology 2023;196:167–173. 36423511 10.1016/j.theriogenology.2022.11.020

[hoag028-B102] Tian J , Yang J, Chen T, Yin Y, Li N, Li Y, Luo X, Dong E, Tan H, Ma Y et al Generation of Human endometrial assembloids with a luminal epithelium using air–liquid interface culture methods. Adv Sci 2023;10:e2301868. 10.1002/advs.202301868 PMC10602567 37635169

[hoag028-B103] Turco MY , Gardner L, Hughes J, Cindrova-Davies T, Gomez MJ, Farrell L, Hollinshead M, Marsh SGE, Brosens JJ, Critchley HO et al Long-term, hormone-responsive organoid cultures of human endometrium in a chemically defined medium. Nat Cell Biol 2017;19:568–577. 28394884 10.1038/ncb3516 PMC5410172

[hoag028-B104] Utsunomiya T , Yao T, Itoh H, Kai Y, Kumasako Y, Setoguchi M, Nakagata N, Abe H, Ishikawa M, Kyono K et al Creation, effects on embryo quality, and clinical outcomes of a new embryo culture medium with 31 optimized components derived from human oviduct fluid: a prospective multicenter randomized trial. Reprod Med Biol 2022;21:e12459. 35431648 10.1002/rmb2.12459 PMC8999156

[hoag028-B105] Vermey B , Buchanan A, Chambers G, Kolibianakis E, Bosdou J, Chapman M, Venetis C. Are singleton pregnancies after assisted reproduction technology (ART) associated with a higher risk of placental anomalies compared with non‐ART singleton pregnancies? A systematic review and meta‐analysis. BJOG 2019;126:209–218. 29740927 10.1111/1471-0528.15227

[hoag028-B106] Wang M , Zhu T, Liu C, Jin L, Fei P, Zhang B. Oviduct-mimicking microfluidic chips decreased the ROS concentration in the in vitro fertilized embryos of CD-1 mice. Biomed Pharmacother 2022;154:113567. 36007278 10.1016/j.biopha.2022.113567

[hoag028-B107] Wiwatpanit T , Murphy AR, Lu Z, Urbanek M, Burdette JE, Woodruff TK, Kim JJ. Scaffold-free endometrial organoids respond to excess androgens associated with polycystic ovarian syndrome. J Clin Endocrinol Metab 2020;105:769–780. 31614364 10.1210/clinem/dgz100 PMC7112974

[hoag028-B108] Xue Y , Ling C, Zheng H, Li K. Supplementation with oviductal EVs from the estrus, metestrus, and diestrus stages improved developmental competence of IVF mouse embryos. Sci Rep 2025;15:23376. 40603993 10.1038/s41598-025-07195-z PMC12222485

[hoag028-B109] Yeung WSB , Ho PC, Lau EYL, Chan STH. Improved development of human embryos in vitro by a human oviductal cell co-culture system. Hum Reprod 1992;7:1144–1149. 1400940 10.1093/oxfordjournals.humrep.a137810

[hoag028-B110] Yucer N , Ahdoot R, Workman MJ, Laperle AH, Recouvreux MS, Kurowski K, Naboulsi DJ, Liang V, Qu Y, Plummer JT et al Human iPSC-derived fallopian tube organoids with BRCA1 mutation recapitulate early-stage carcinogenesis. Cell Rep 2021;37:110146. 34965417 10.1016/j.celrep.2021.110146 PMC9000920

[hoag028-B111] Zagers MS , Laverde M, Goddijn M, de Groot JJ, Schrauwen FAP, Vaz FM, Mastenbroek S. The composition of commercially available human embryo culture media. Hum Reprod 2025;40:30–40. 39585967 10.1093/humrep/deae248 PMC11700899

[hoag028-B112] Zhang C , Tian H, Yin X, Dong X, Wang N, Zhang Y, Wang J, Song J, Li C, Chen Z-J et al Crosstalk between H3K4me3 and oxidative stress is a potential target for the improvement of ART-derived embryos. Nat Commun 2025;17:852. 41402350 10.1038/s41467-025-67560-4 PMC12828018

[hoag028-B113] Zhang Y , Zhao R, Yang C, Song J, Liu P, Li Y, Liu B, Li T, Yin C, Lu M, et al Human receptive endometrial organoid for deciphering the implantation window. eLife 2024;12:RP90729. 10.7554/eLife.90729 PMC13043174 41920166

[hoag028-B114] Zhu P , Guo H, Ren Y, Hou Y, Dong J, Li R, Lian Y, Fan X, Hu B, Gao Y et al Single-cell DNA methylome sequencing of human preimplantation embryos. Nat Genet 2018;50:12–19. 29255258 10.1038/s41588-017-0007-6

